# Association of Sonic Hedgehog with the extracellular matrix requires its zinc-coordination center

**DOI:** 10.1186/s12860-021-00359-5

**Published:** 2021-04-16

**Authors:** Carina Jägers, Henk Roelink

**Affiliations:** University of California, Department of Cell and Molecular Biology, Berkeley, CA USA

**Keywords:** Shh signaling, Zinc metallopeptidases, Extracellular matrix, BacHh

## Abstract

**Background:**

Sonic Hedgehog (Shh) has a catalytic cleft characteristic for zinc metallopeptidases and has significant sequence similarities with some bacterial peptidoglycan metallopeptidases defining a subgroup within the M15A family that, besides having the characteristic zinc coordination motif, can bind two calcium ions. Extracellular matrix (ECM) components in animals include heparan-sulfate proteoglycans, which are analogs of bacterial peptidoglycan and are involved in the extracellular distribution of Shh.

**Results:**

We found that the zinc-coordination center of Shh is required for its association to the ECM as well as for non-cell autonomous signaling. Association with the ECM requires the presence of at least 0.1 μM zinc and is prevented by mutations affecting critical conserved catalytical residues. Consistent with the presence of a conserved calcium binding domain, we find that extracellular calcium inhibits ECM association of Shh.

**Conclusions:**

Our results indicate that the putative intrinsic peptidase activity of Shh is required for non-cell autonomous signaling, possibly by enzymatically altering ECM characteristics.

**Supplementary Information:**

The online version contains supplementary material available at 10.1186/s12860-021-00359-5.

## Background

The *Hedgehog* (*Hh*) gene was first identified in the *Drosophila melanogaster* screen performed by Christiane Nüsslein-Volhard and Eric Wieshaus in the late 1970s [1]. Like other segment polarity genes found in this screen, *Hh* genes are widely conserved among animals, and mammals have three Hh paralogs that play roles in development [2]. Like all other Hhs, Sonic Hedgehog (Shh) is synthesized as a pro-protein that undergoes autoproteolytic cleavage mediated by the C-terminal part yielding an N-terminal part (ShhNp) that is the active ligand. Structural analysis of ShhN revealed its similarity to zinc-peptidases and Shh coordinates a zinc ion with residues H141, D148, and H183 [3]. The notion that Shh signaled through a peptidase activity was quickly rejected by demonstrating that mutation of a critical residue involved in catalysis (E177) did not impair the ability of Shh to activate the Hh response [4], and consequently the zinc coordination center of Shh is often referred to as its “pseudo active” site [5, 6]. Still, a role for the zinc coordination center is supported by the finding that Shh-E177A is unable to mediate non-cell autonomous long-range signaling from the notochord to the overlying neural plate [7]. Perhaps unsurprisingly, the zinc-coordination motif is found mutated in some individuals with the Shh signaling-related birth defect holoprosencephaly [8, 9], further indicating that the zinc-coordination center of Shh is important for normal function. This is consistent with structures of Shh complexed with its receptor Patched1 (Ptch1), showing that the N-terminal 22 residues of Shh that are not part of the zinc-coordination motif, mediate binding to Ptch1 [10–12] and suffice to regulate Ptch1 activity [13].

Some bacterial species have conserved genes coding for peptidases that coordinate zinc and calcium identically to Shh [14, 15]. These bacterial peptidases (members of the M15A subfamily of zinc D-Ala-D-Ala carboxypeptidases) cleave murein peptidoglycans, suggesting that Shh too might cleave a glycan-modified protein, possibly a matrix heparan sulfate proteoglycan (HSPGs). HSPG are an integral part of the extracellular matrix (ECM) and play in important role in the transport and presentation of several morphogens, including Hhs [16]. Several HSPGs bind Shh and can both negatively and positively affect the Shh response [17–20]. Furthermore, mutations in *Ext1* and *− 2* coding for glycosyltransferases that catalyze glycosaminoglycan addition to the core proteins, disrupt Hh signaling in vertebrates [20, 21] and insects [22].

By mutating residues in the zinc-coordination center that are conserved between bacterial Hh-like peptidases and Shh, we provide evidence that this center is required for the association of ShhN to the ECM and for non-cell autonomous signaling. Release of Shh into the ECM is enhanced in the presence of μM amounts of zinc indicating that this ion is an agonist of Shh. The ECM-associated Shh is active in signaling, indicating that the zinc-coordination center of Shh mediates its release into the ECM to facilitate non-cell autonomous signaling, possibly through an intrinsic metallopeptidase activity of Shh.

## Results

### ShhN associates with the extracellular matrix

**Fig. 1:**
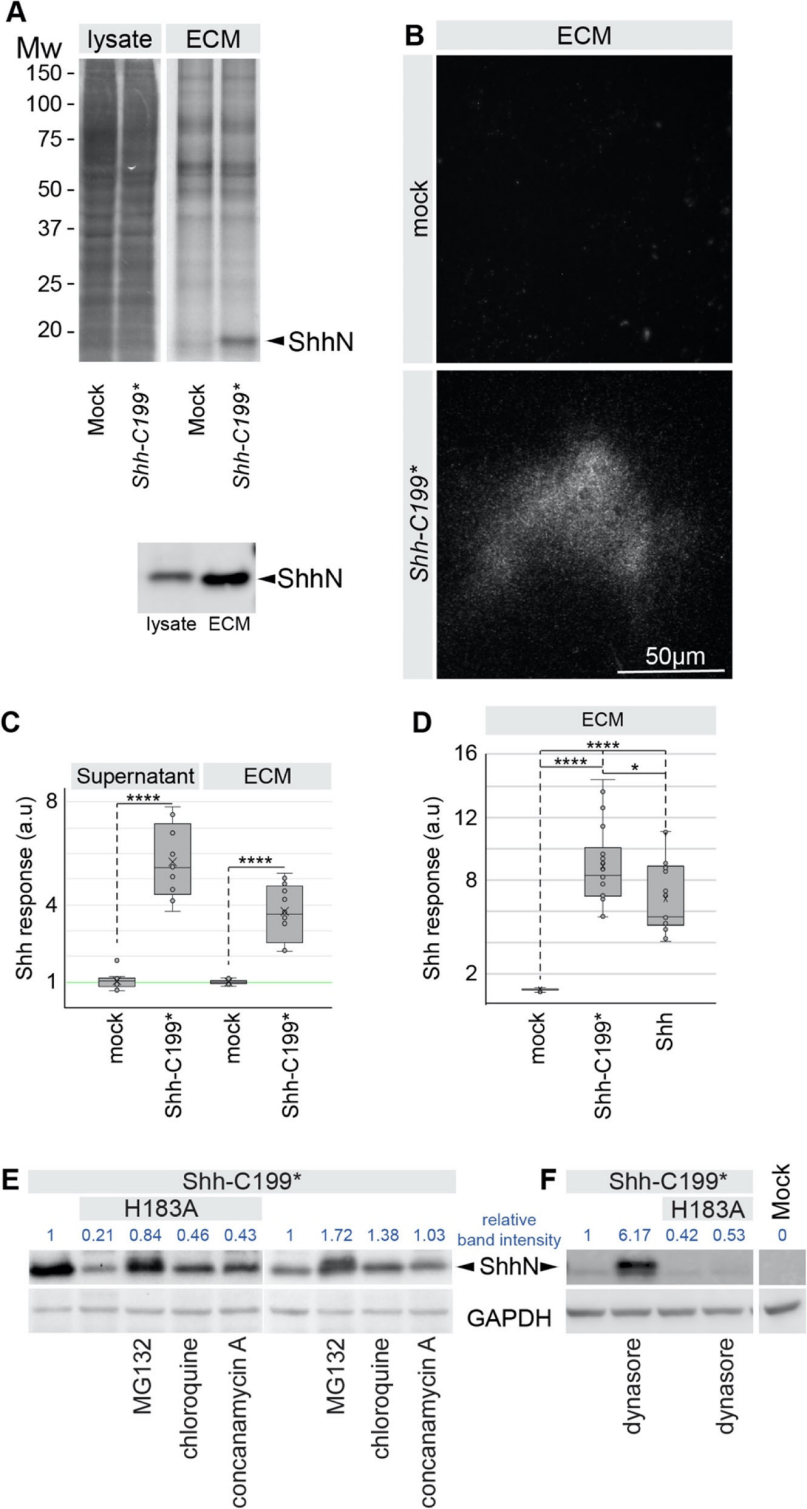
Active ShhN associates with the extracellular matrix. **a** Lysate and ECM deposited by mock and Shh-C199*-transfected Hek293t cells analyzed by SDS-PAGE/SYPRO-Ruby staining and on a Western Blot (H2 α-ShhN). Equal fractions of the total cell lysate and ECM were analyzed. ShhN is indicated. **b** Shh-C199*-transfected Hek293t cells were plated on glass slides and removed after 24 h. The slides were stained with mAb5E1, showing the presence of ShhN. Scale bar is 50 μm. **c** Supernatant and ECM conditioned by Shh-C199*-transfected Hek293t cells. LightII cells were either grown on mock or ShhN conditioned ECM. Cells grown on ECM deposited by mock transfected cell were grown in the absence or presence of mock or Shh-C199* conditioned supernatant. Box and whisker plots, *n* ≥ 3. *p < 0.05, ****p < 0.0001. **d** Hh response in LightII cells grown on the decellularized ECM of mock-, Shh-C199*-, or *Shh*-transfected Hek293t cells. ****p < 0.0001. **e** Western blot analysis of Hek293t cells transfected with the indicated Shh mutants. 100 nM MG-132 (proteasome inhibitor), 100 nM Chloroquine and 100 nM Concanamycin A (inhibitors of endosome acidification) were assessed for their ability to affect Shh accumulation. Immune reactive signals are quantified and normalized to the untreated and Shh-C199* transfected condition. **f** Western blot analysis of Hek293t cells transfected with the indicated Shh mutants, and the effects of the dynamin inhibitor Dynasore (50 μM) was assessed for its effect on Shh accumulation. Immune reactive signals are quantified and normalized to the untreated and Shh-C199* transfected condition. Full-length gels blots are presented in Supplementary Figure 3

Due to its very high sequence similarity to bacterial murein peptidases [15], a conceivable function for the Shh zinc-coordination center could be to modify proteoglycans, thereby affecting its extracellular matrix (ECM) association. Cultured cells condition their substrate with functional ECM proteins like fibronectin and collagen, at least some of which is retained by the substrate after removal of the cells [23]. Shh has a Cardin-Weintraub motif that mediates binding to heparan sulfate in the ECM [24, 25], and we assessed ECM-bound Shh in the fraction of macromolecules that remain on the tissue culture plate after non-lysing cell removal. Hek293t cells were transfected with *Shh* (mutant) constructs and after 2 days the cells were removed by washing with PBS and mild agitation. The cells were then collected and lysed with RIPA buffer for protein gel and Western Blot analysis. The tissue culture dishes were extensively washed with PBS and remaining material was collected in hot SDS using a scraper for further analysis [23]. We will refer to this as the ECM fraction. Using gel electrophoresis followed by SYPRO Ruby protein staining, we detected overall fewer proteins in the fraction remaining in the tissue culture dish (ECM) compared to the cell-only fraction (lysate) with a pattern distinct from the lysate indicating recovery of extracellular molecules (Fig. 1a). More Shh could be detected in the ECM than in the cell-only fraction of Shh-C199* transfected Hek293t cells (Fig. 1b), showing that entry of ShhN into the ECM is robust. Here, we are focusing on the association of Shh with the ECM, and in order to circumvent the complexities of Shh maturation and secretion [26], we used Shh-C199* (ShhN). This form of Shh is active and secreted independent of Disp1 function [27], and lacks the C-terminal sterol modification. We found that ShhN could readily be detected in the ECM extracted from decellularized tissue culture plates (Fig. 1b). Visualizing Shh-C199* by staining of the decellularized plates with the anti-Shh mAb5E1, “footprints” of Shh producing cells were observed (Fig. 1b). Shh-C199* is commonly thought of as a “soluble” protein and also accumulates in the supernatant of ShhN-producing cells. The non-homogeneous association of Shh with the ECM in apparent “footprints” of the Shh-C199*-expressing cells indicates that upon secretion Shh enters adjacent ECM directly and is not first released into the supernatant, which would result in a more homogenous distribution of the protein across the ECM. Shh-responsive LightII cells plated on the decellularized and Shh-conditioned ECM showed that it is able to elicit a transcriptional Hh pathway response similar to that of ShhN-conditioned medium (Fig. 1c). Furthermore, wild-type Shh bound to the ECM also activated the Hh pathway response in LightII cells (Fig. 1d).

### Mutations in the zinc-coordination motif reduce the stability of Shh-C199*

Mutating the residues directly involved in the coordination of zinc (H141, D148, H183) are obvious candidates to assess a role for the zinc-coordination center. However, Shh mutants in the zinc coordination motif could barely be detected as the N-terminal processed form (ShhNp) on Western Blots, despite normal detection of the Shh pro-protein [28]. We and others [9] initially incorrectly interpreted this as a failure of auto-processing. However, testing these mutants in Shh-C199* revealed that even when auto-processing is circumvented, these mutants are still detected at lower levels. To assess if these mutants are unstable, we added protease inhibitors. Addition of the proteasome inhibitor MG132 [29] or inhibitors of endosome acidification (chloroquine or concanamycin A) resulted in ShhN accumulation of Shh-C199*/H183A (ShhN/H183A), possibly indicating a misfolded protein-induced degradation of this mutant via the proteasome and to a smaller extend the lysosome (Fig. 1e). The Dynamin inhibitor Dynasore (that inhibits endocytosis) [30] causes strong accumulation of Shh-C199*, but not of Shh-C199*/H183A, further indicating that the destabilization of Shh-C199*/H183A occurs before it reaches the plasma membrane (Fig. 1f). We found that other zinc coordination mutations as well as several holoprosencephaly-associated point mutations in Shh cause its destabilization, indicating a role for increased ShhN degradation in this birth defect [28]. In general, we will not use these mutants with a reduced half-life.

### The zinc-coordination center of Shh is required for association with the ECM

**Fig. 2:**
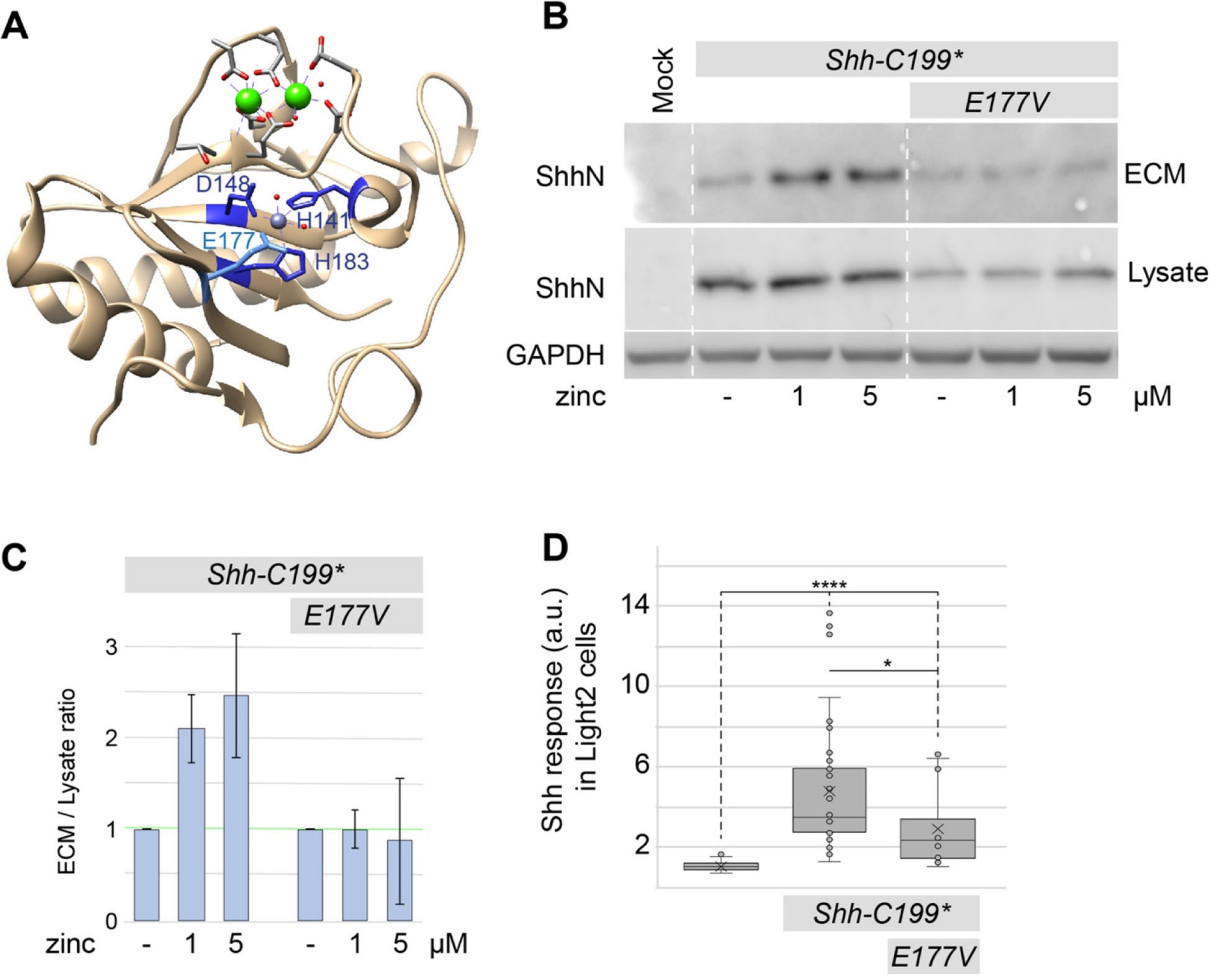
ECM-association of ShhN requires zinc and the catalytic E177V residue. **a** Diagram of the Shh structure (PDB: 3D1M) with relevant residues indicated. **b** Western blot analysis of the lysate and ECM of Hek293t cells transfected with Shh-C199* and Shh-C199*/E177V and cultured in DMEM containing 0.18 mM calcium and the indicated concentrations of zinc. Uncropped blots can be found in the supplement. **c** Quantification of band intensities shown in C as a fraction of ECM over lysate, normalized to the respective mock treatment to better illustrate the effects of zinc treatment. *n* = 2. **d** Hh response in LightII cells grown on the conditioned and decellularized ECM of Hek293t cells transfected with Shh-C199* or Shh-C199*/E177V. n ≥ 3, *p < 0.05, ****p < 0.0001. Full-length blots are presented in Supplementary Figure 4

BacHh belongs to a family of metallopetidases that coordinate zinc, and consistent with the absolute conservation of the zinc-coordination center, zinc was found in the catalytic cleft of Shh (Fig. 2a, grey sphere). We hypothesized that occupancy of the zinc-coordination center is required for normal Shh function. The K_d_ for zinc binding to Shh in the absence of calcium appears to be low [31], but DMEM tissue culture medium has no added zinc and is thus expected to have only very small amounts of it. While the amount of protein in the lysate of Hek293t cells transfected with Shh-C199* remained relatively unchanged with increasing zinc concentrations, the amount of ECM-bound Shh-C199* increased approximately two and a half times with an EC_50_ between 0.1 and 1 μM zinc (Fig. 2b, c). This indicates that there is little effect of zinc on Shh synthesis and intracellular stability, but that occupancy of the zinc coordination center enhances ECM association. Divalent copper and magnesium failed to increase the amount of ShhN in the ECM (Fig. S2). Calcium, however, did have an effect consistent with its ability to bind to ShhN, and is further addressed below.

Besides the zinc coordinating residues, the glutamic acid residue at position 177 (E177, mouse numbering) is well-conserved and in close proximity to the zinc-coordinating residues (Fig. 2a). In Shh-related peptidases the E177 equivalent is required for catalytic activity as it strips a proton from water yielding a reactive hydroxide. The mutants Shh-C199*/E177A and −/E177V are predicted to be able to coordinate zinc but lack the putative catalytic activity. Unlike the zinc coordination mutants, we found the Shh-C199*/E177A and −/E177V mutants to be stable in the lysate (Fig. 2b). Shh-C199*/E177V in decellularized ECM activated the Hh response in LightII cells to a smaller extend than Shh-C199* (Fig. 2d). This was likely not caused by lower protein amounts of Shh-C199*/E177V in the ECM, as we found that the amount of Shh-C199*/E177V in the ECM was similar to that of Shh-C199* under low zinc concentrations but failed to further accumulate in the ECM under increasing zinc concentrations. This demonstrates that ShhE177 is required for ECM association (Fig. 2b, c), and importantly that the zinc effects are not primarily mediated by a Shh-independent zinc sensitive event. These observation supports the notion that a catalytic activity intrinsic to Shh is required for its association with the ECM.

### Mutations in additional conserved residues in the zinc-coordination motif of Shh affect association with the ECM

**Fig. 3:**
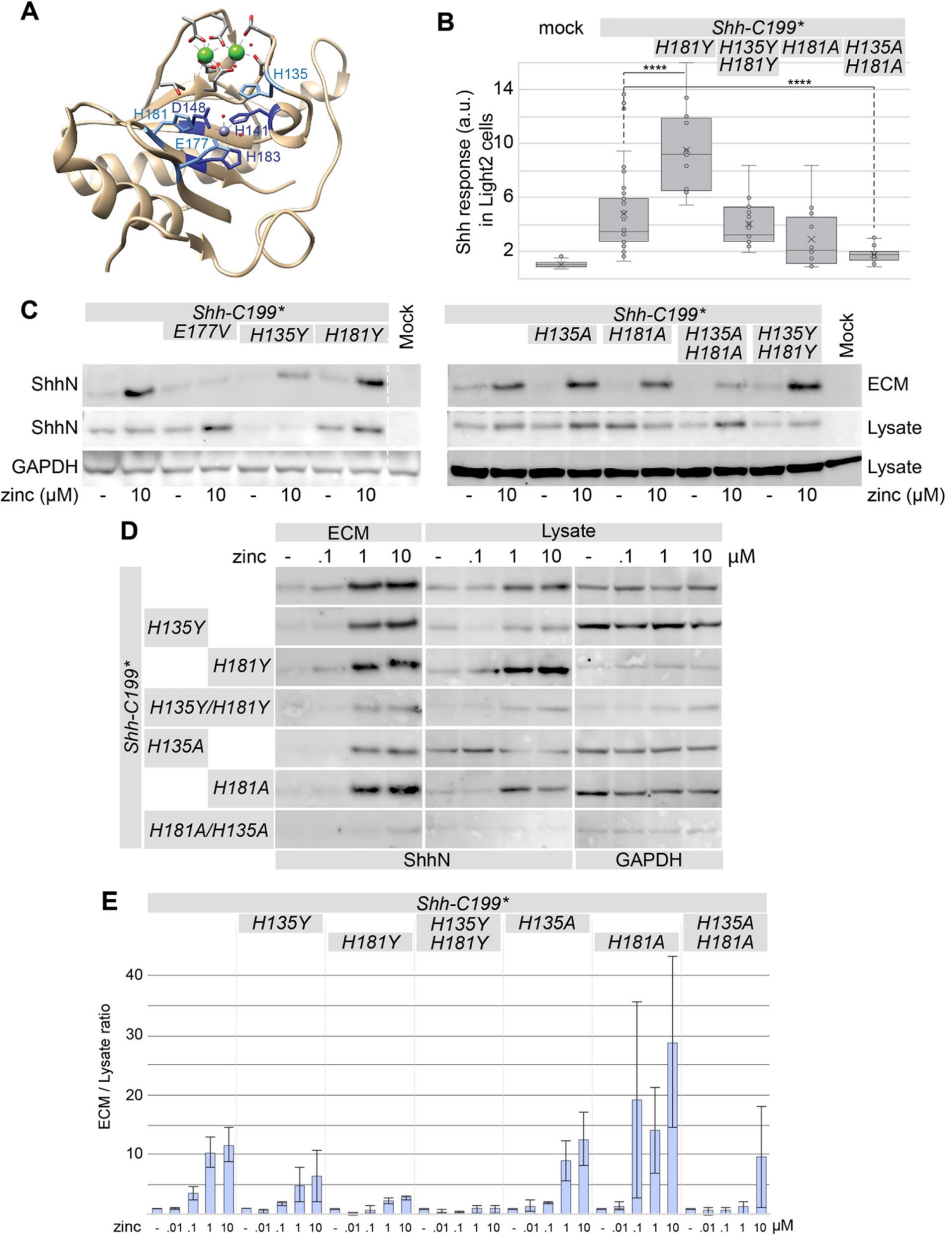
Stacking histidines of the zinc coordination center affect ECM association. **a** Diagram of the Shh structure (PDB: 3D1M) with relevant residues indicated. **b** Hh response in LightII cells grown on the conditioned and decellularized ECM of Hek293t cells transfected with the indicated Shh-C199* constructs. *n* ≥ 2, *p < 0.05, ****p < 0.0001. **c** The effect of mutations of the transition state-stabilizing residues H135 and H181 to alanine (A) or tyrosine (Y) on the zinc-dependent accumulation in the ECM was analyzed on a Western Blot of the extracted ECM from transfected Hek293t cells cultured in 0.18 mM calcium with or without 10 μM zinc. **d** zinc dose-response analysis of H135 and H181 mutations assessed by Western blot of the lysate and ECM of Hek293t cells transfected with the indicated mutants and cultured in 0.18 mM calcium and increasing concentrations of zinc (0.1, 1, 10 μM). Uncropped blots can be found in the supplement. **e** Quantification of band intensities shown in D as a fraction of ECM over lysate, normalized to the respective mock treatment. n = 2. Uncropped, full-length blots are presented in Supplementary Figure 5

A second group of conserved residues in the zinc-coordination motif are two histidine residues with stacking sidechains, H135 and H181 (Fig. 3a). These two histidine residues are conserved between Shh and BacHhs, but either one can be a tyrosine residue in M15A peptidases, and a tyrosine residue is present in the position homologous to H181 in butterfly and moth Hhs (e.g. NCBI PCG69308.1). We mutated either or both histidine residues 135/181 into alanine or tyrosine residues (Shh-C199*/H135Yor*A*, Shh-C199*/H181Yor*A*) and found that these forms of Shh process normally and are stable in the lysate (Fig. 3c). Substituting one or two histidines with tyrosines had little effect on the mutant’s ability to elicit a Hh response in LightII cells from conditioned ECM (Fig. 3b). Alanine substitutions reduced the Hh response in the LightII compared to Shh-C199* (Fig. 3b). Similar to Shh-C199*/E177V, the reduced Hh response coincided with lower protein levels in the ECM. Mutants with one or two tyrosine substitutions as well as single alanine substitutions could be rescued under high zinc conditions (1, 10 μM) suggesting that tyrosine residues, as they are found in butterfly and moth Hhs, are largely synonymous mutations. Only Shh-C199*/H135A/H181A poorly associated with the ECM in the presence of zinc. We found that all H135 and H181 mutants have a similar EC_50_ for zinc (Fig. 3d and quantified in Fig. 3e), consistent with the notion that these residues are not directly involved in zinc coordination. Together with Shh-C199*/E177V, our findings using Shh-C199*/H135A/H181A further support the notion that the zinc-coordination center of Shh is required for ECM association.

### The peptidase domain of a BacHh is unable to facilitate ECM association

**Fig. 4:**
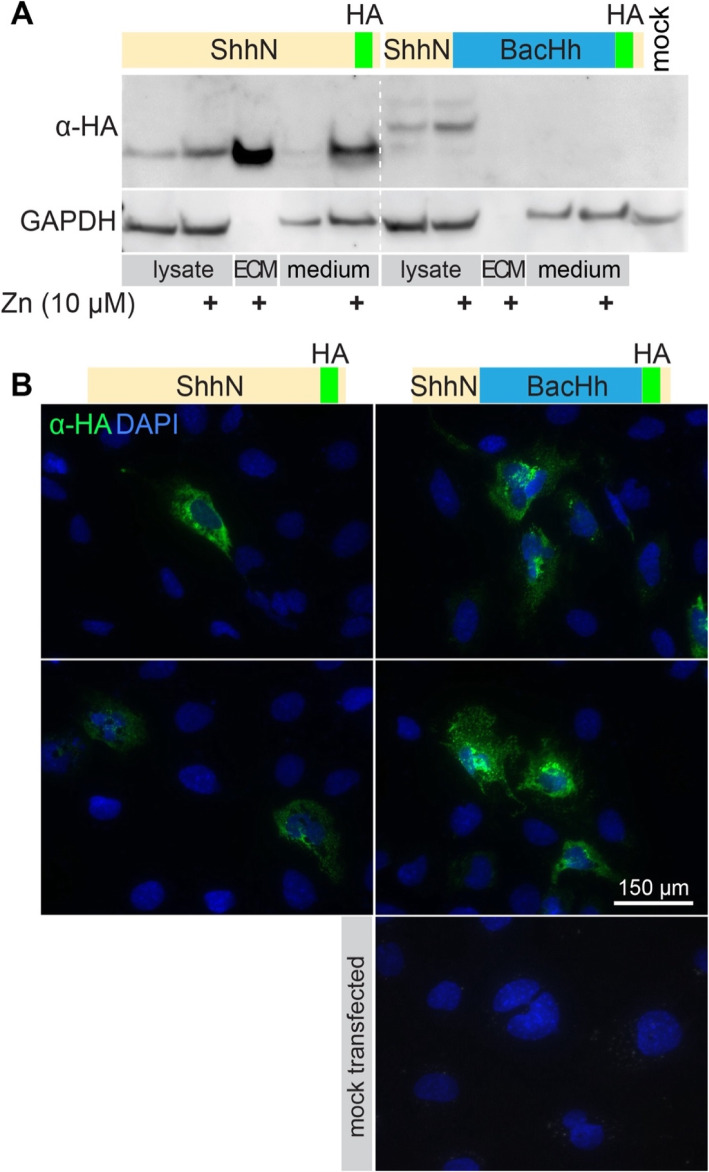
The zinc-coordination domain of BacHh is not sufficient for association with the ECM. **a** Western Blot analysis of the lysate, ECM, and supernatant of *ShhN-HA* or *Shh-BacHh-HA* (diagrams) transfected Hek293t cultured in the indicated zinc concentrations. Uncropped blots can be found in the supplement. **b** Detergent-free live staining with an α-HA antibody (3F10) of transfected *Ptch1*^LacZ/LacZ^;*Ptch2*^−/−^*;Boc*^−/−^;*Cdo*^−/−^;*Gas1*^−/−^ cells. Nuclei were stained with DAPI. Uncropped, full-length blots are presented in Supplementary Figure 6

The protein sequences of bacterial Hhs (BacHh) are highly similar Shh and all residues of the zinc coordination motif are identical. As BacHhs are predicted to be peptidoglycan peptidases we tested if this bacterial peptidase activity could substitute for the putative peptidase activity intrinsic to ShhN. The conservation between BacHhs and Shh involves the calcium and zinc binding motifs, but not the N-terminal domain that binds to Ptch1 and Heparan Sulfate [24], nor the 10 amino acids that follow this domain in ShhN. Therefore, we made a construct coding for a chimeric protein consisting of the N-terminal 65 residues of Shh, the conserved calcium and zinc binding motifs of *Bradyrhizobium paxllaeri* BacHh (codon optimized for expression in mammalian cells), followed by an HA tag replacing the bacterial stop codon, followed by the last 10 residues of Shh up to G198 (Shh/BacHh^HA^, Fig. 4a diagram). As a control, we positioned an HA tag at the same distance (10 residues) from the C-terminus of Shh-C199* (Shh^HA^-C199*). We found that Shh^HA^-C199* behaved indistinguishable from Shh-C199* and entered into the ECM and the medium in a zinc-dependent manner (Fig. 4a). In contrast, although readily detected in the lysate, no Shh/BacHh^HA^ was detected in the ECM or the medium (Fig. 4a). We detected similar amounts of GAPDH in the medium as in the cell lysate. GAPDH is commonly used as a loading control for intracellular proteins but has also been described as a soluble form found in the extracellular compartment [32]. Cell lysis in the tissue culture as an explanation for GAPDH in the medium seems unlikely, as no Shh/BacHh^HA^ was detectable in the soluble fraction. As ShhN can be internalized by several Shh-binding proteins, [33–35], we assessed whether the chimeric proteins accumulates on the outside of cells with detergent-free, live staining with an α-HA antibody prior to fixation of transfected receptor-less (*Ptch1*^LacZ/LacZ^;*Ptch2*^−/−^*;Boc*^−/−^;*Cdo*^−/−^;*Gas1*^−/*−*^) fibroblasts. We found no difference in staining between Shh^HA^-C199* and Shh/BacHh^HA^, indicating that Shh/BacHh^HA^, similarly to Shh^HA^-C199*, is being trafficked to the plasma membrane (Fig. 4b). It is not, however, being released from the cell, indicating that the bacterial zinc-coordination domain is not sufficient for entry into the ECM. *Bradyrhizobium paxllaeri* BacHh presumably lacks the specificity for an ECM binding partner that is recognized by Shh. These results suggest that the observed presence of Shh and ShhN in the decellularized tissue culture plate is due to a precise property of the Shh rather than simple cell lysis or ShhN-containing cell debris.

### Shh-C199* mutants unable to bind calcium remain sensitive to zinc

**Fig. 5:**
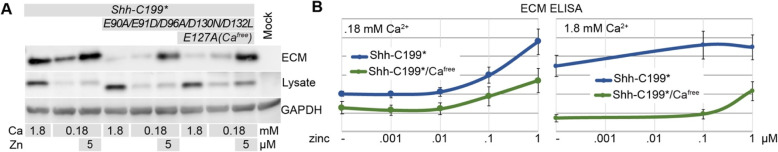
Calcium alters the sensitivity of Shh to zinc. **a** Western blot analysis of lysates and ECM of Hek293t cells transfected with Shh-C199* and Shh-C199*/E90A/E91D/D96AD130N/D132L, or Shh-C199* and Shh-C199*/E90A/E91D/D96A/E127A/D130N/D132L (*Ca*^*Free*^), cultured in the presence of 0.18 or 1.8 mM calcium, and in the absence or presence of 5 μM added zinc. **b** ECM-associated Shh-C199*N or Shh-C199*/Ca^Free^ deposited by transfected Hek293t cells was assessed by ELISA in the presence of .18 mM calcium (left panel) or 1.8 mM calcium (right panel) and increasing zinc concentrations as indicated. Shown are means and standard errors, *n* = 6. Uncropped, full-length blots are presented in Supplementary Figure 7

The overall structure of ShhN and the BacHhs indicate that they consist of a regulatory calcium-binding and a catalytic zinc coordinating center [14], making up most of ShhN outside the extreme N-terminal Ptch1-binding domain. With the exception of BacHhs, bacterial M15A metallopeptidases lack the Hh/BacHh-type calcium coordination center, and this part is thus unlikely to be required for catalytic function per se. We made a Shh-C199* mutant that lacked all calcium-coordinating residues (Shh-C199*/E90A/E91D/D96A/E127A/D130N/D132L, Shh-C199*-Ca^Free^) and this form of Shh should be unable to bind calcium. After transfection, more ShhN was detected in lysates of cells cultured in the presence of higher calcium levels, but that was also observed in the Shh-C199*-Ca^Free^ expressing cells, and thus unlikely a direct effect of calcium on Shh (Fig. 5a). Increased amounts of ShhN in the lysate at higher calcium concentrations complicated the interpretation of the effects of calcium on ShhN accumulation in the ECM. Sill, while ShhN accumulation in the ECM varied with calcium concentrations, that of the Shh-C199*-Ca^Free^ mutant remained at the same level, indicating that this mutant is insensitive to extracellular calcium as measured by ECM association (Fig. 5a).

One possible mechanism of calcium regulating the transition from a cell- to an ECM-bound state would be by affecting zinc coordination, thereby changing its K_d_ for zinc. We therefore tested if the EC_50_ of zinc is different under high (1.8 mM, the concentration in regular DMEM) and low (0.18 mM, the lowest concentration the cultured cells appeared normal) calcium. Under low calcium conditions, the addition of 5 μM zinc to the medium resulted in increased accumulation of ShhN in the ECM both of Shh-C199*-Ca^Free^ and Shh-C199* (Fig. 5a). This indicates that Shh-C199*-Ca^Free^ is still active, and supports the notion that calcium binding is not required for Shh distribution. E127 is located at the interface between the calcium and zinc-binding centers of Shh, and we tested if restauration of this residue in Shh-C199*-Ca^Free^ affects ECM localization but found little or no difference (Fig. 5a). To better quantify the effect of calcium and zinc on Shh-C199* and Shh-C199*-Ca^Free^ in their ability to accumulate in the ECM we used an indirect ELISA protocol directly on the decellularized ECM. Hek293t cells were cultured and transfected in 96 well plates and washed off with PBS to allow for subsequent detection of ShhN with HRP-linked antibodies. Under low calcium conditions we found that the response to increasing zinc concentrations was similar between Shh-C199* and Shh-C199*-Ca^Free^ (Fig. 5b). For both, the EC_50_ for zinc appeared to be around 0.1 μM. Instantiated in Fig. 5a and quantified over multiple experiments in Fig. 5b, it appears that Shh-C199*-Ca^Free^ is less efficient in entering the ECM than Shh-C199*. This effect was more profound in the presence of 1.8 mM calcium, and much more Shh-C199* was detected in the ECM than Shh-C199*-Ca^Free^ in the absence of added zinc. The addition of zinc had a bigger effect on Shh-C199*-Ca^Free^ than on Shh-C199*. These results indicate that Shh-C199*-Ca^Free^ behaves similarly in high and low calcium and resembles Shh-C199* under low calcium. Thus, whereas the behavior of Shh-C199* changes as a function of calcium, that of Shh-C199*-Ca^Free^ does not, indicating that binding of calcium to Shh alters its intrinsic properties as measured by its ECM association. The calcium concentration in the endoplasmic reticulum and the Golgi apparatus is variable, with values ranging from .2-1 mM [36]. These concentrations that Shh encounters in these organelles are within the range in which we observe changes in zinc sensitivity of ShhN. This is consistent with the notion that the activity of ShhN could be regulated by calcium.

### Distribution of cholesterol-modified ShhNp in the ECM differs from cholesterol-unmodified ShhN but remains zinc sensitive

**Fig. 6:**
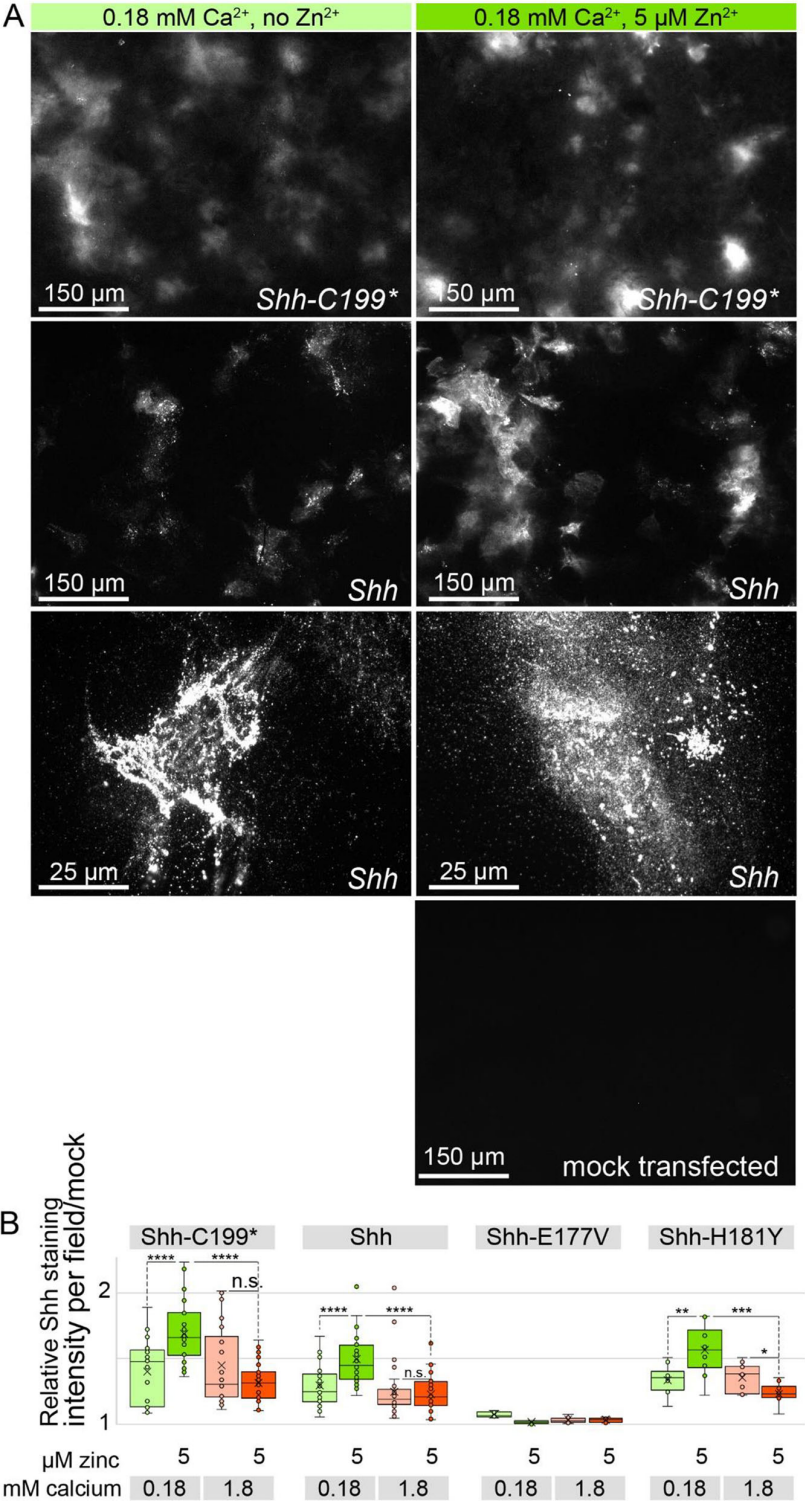
Cholesterol-modified ShhNp associates with the ECM in a zinc and peptidase-dependent manner. **a** In situ staining with anti ShhN mAb5E1 of ECM deposited by *Ptch1*^*LacZ/LacZ*^;*Ptch2*^*−/−*^*;Boc*^*−/−*^;*Cdo*^*−/−*^;*Gas1*^*−/−*^ cells that were transfected with *Shh* or Shh-C199* in the presence or absence of 5 μM zinc and presence of 0.18 mM calcium. **b** Quantification of ECM-bound Shh shown in A. Box and whisker plots of mean fluorescence intensity per image of 10 microscope fields per experiment was measured in ImageJ and normalized to the ECM of mock transfected cells. *n* = 3, ****p < 0.0001)

While ShhN could readily be detected in the ECM of Hek293t cells, ShhNp was poorly detectable on Western Blots of the ECM fraction of Hek293t cells. We therefore turned to staining of decellularized ECM of our line of fibroblasts lacking Shh binding partners (*Ptch1*^LacZ/LacZ^;*Ptch2*^−/−^*;Boc*^−/−^;*Cdo*^−/*−*^;*Gas1*^−/−^) that were found to accumulate ShhNp in the ECM to comparable levels as ShhN, presumably due to a failure to re-internalize ShhN(p) (Fig. 6a and b). Staining for Shh on decellularized plates showed that ShhN was present in small puncta that gave a cloudy appearance at lower magnifications, whereas cholesterol-modified ShhNp was detected in larger puncta in more restricted areas (Fig. 6a). 5 μM zinc increased ShhN and ShhNp association with the ECM as measured by fluorescence intensity across the entire image area (Fig. 6b).

The effects of zinc on Shh distribution in the presence of 1.8 mM calcium was much less pronounced, further supporting the finding that high calcium negatively affects the zinc-dependent activity of Shh.

To assess if the observed effects require the zinc-coordination center of Shh we tested Shh-E177V, and -H181Y for their ability to associate with the ECM. Consistent with the biochemical observations using Shh-C199* (Figs. 1, 2, 3), we could barely visualize ShhE177V in the ECM (Fig. 6b). In contrast, ShhH181Y distribution into the ECM was indistinguishable from Shh, further indicating that this “butterfly version” of Shh is functional (Fig. 6b).

### Mutations in the putative peptidase domain reduce non-cell autonomous signaling

**Fig. 7:**
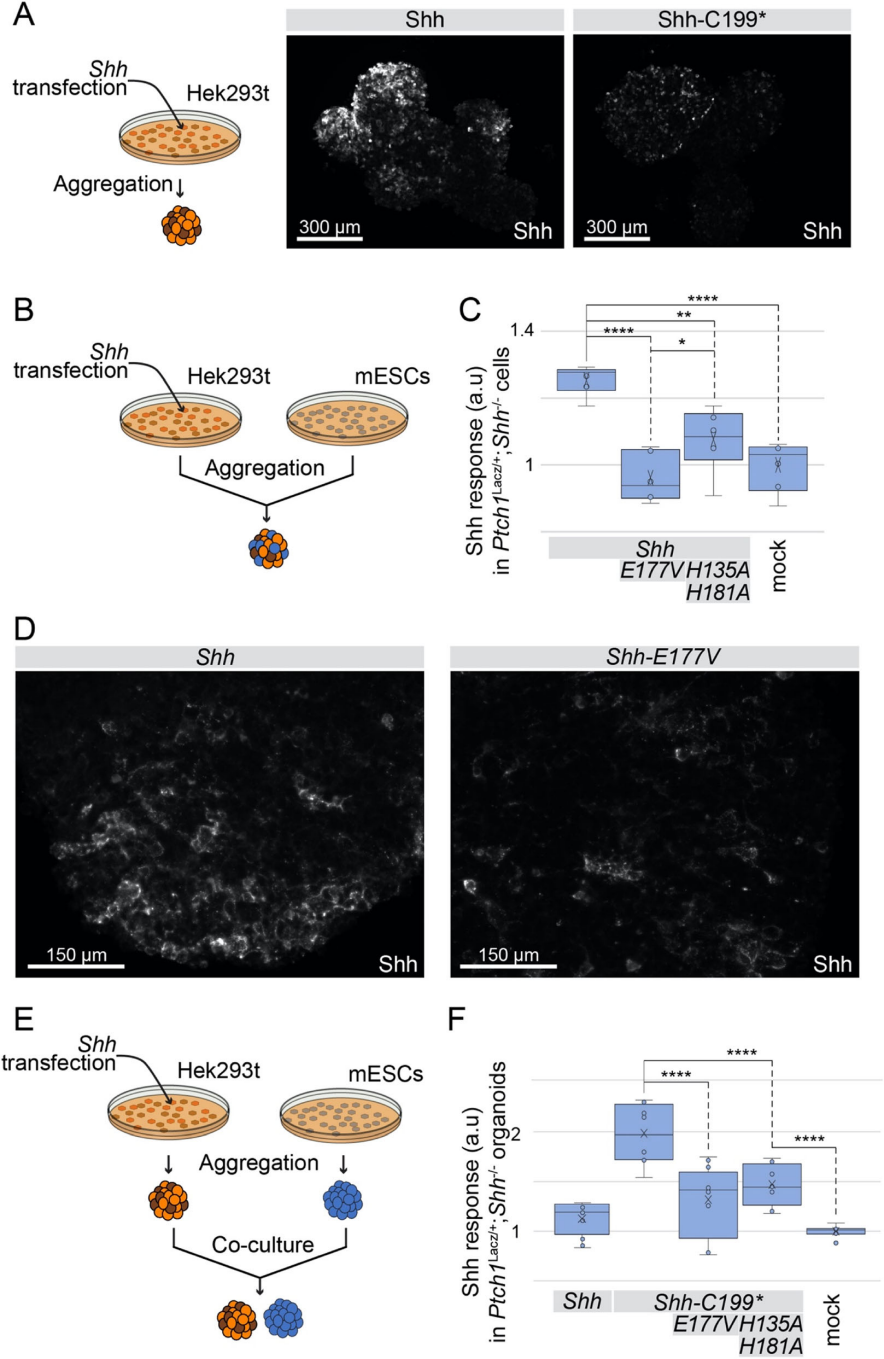
The zinc coordination center of Shh is required for long-range signaling. **a** Experimental setup and immunofluorescent staining of Shh-expressing Hek293t aggregates. Hek293t cells were transfected with *Shh* or Shh-C199*, washed off the culture dish with PBS and placed on a rotating platform in DMEM without serum for 2 days, followed by live staining with 5E1. Shades of brown represent differences in transfection efficiency. **b** Experimental setup: Hek293t cells were transfected with *Shh*-constructs, washed off the culture dish with PBS, and mixed with mESC *Ptch1*^LacZ/+^*;Shh*^−/−^ reporter cells in a 1:4 ratio. Chimeric aggregates were then formed on a rotating platform in the absence of serum. The Ptch1:LacZ expression was measured after 3 days and normalized to total protein content. **c** Ptch1:LacZ expression in chimeric aggregates in response to the indicated versions of Shh expressed by Hek293t cells. Box-and-Whisker plot, n = 2. *p < 0.05, **p < 0.01, ****p < 0.0001. **d** 5E1 live staining of Shh in the chimeric aggregates described in B and C. **e** Experimental setup: Hek293t cells were transfected with *Shh*-constructs, washed off the culture dish with PBS, and incubated on a rotating platform. Separate mESC *Ptch1*^LacZ/+^*;Shh*^−/−^ reporter aggregates were added to the dish in a 1:4 ratio and Ptch1:LacZ expression in mESCs was measured after 3 days. **f** Ptch1:LacZ expression normalized to total protein in mESC aggregates co-cultured with Shh-expressing Hek293t aggregates. Box-and-Whisker plot, *n* = 4, ****p < 0.0001

To test if the increased accumulation of Shh in the ECM is correlated with the non-cell autonomous signaling efficacy, we assessed the signaling activity of ShhN expressing cells embedded in three-dimensional cell aggregates of responding cells (Fig. 7a). Live staining with 5E1 prior to fixation and cell permeabilization showed that ShhNp and ShhC199* were detected around a large proportion of cells in transfected Hek293t aggregates. In mixed aggregates consisting of 80% Hh-responsive *Ptch1*^LacZ/+^*;Shh*^−/−^ mouse embryonic stem cell (mESC) reporter cells and 20% transfected Hek293t cells (Fig. 7b), we observed that both Hek293t-derived ShhNp and Shh-C199* induced the Shh response (Fig. 7c). Shh peptidase mutants (Shh-E177V and Shh-H135A/H181A) failed to elicit a Hh-response, further supporting the notion that the peptidase activity is required for non-cell autonomous signaling (Fig. 7c). The pattern of Shh extracellular distribution was similar for Shh and Shh-E177V as seen in detergent-free live staining with 5E1, although slightly weaker staining for Shh-E177V was observed (Fig. 7d). In a more stringent non-cell autonomous assay, aggregated of transfected Hek293t cells were co-cultured with 3D spinal cord organoids (SCOs) derived from the mESC *Ptch1*^LacZ/+^*;Shh*^−/−^ reporter cells (Fig. 7e). Whereas the Hh response to ShhNp was below detection level in this assay, Shh-C199* induced the Shh response in reporter cells (Fig. 7f), likely via a soluble form of ShhN. Putative peptidase mutants failed to show any activity in this assay, further supporting the idea that an intact zinc-coordination center of Shh is necessary to mediate the release from Shh-producing cells and consequently, the facilitation of long-range signaling.

## Discussion

Here, we provide evidence for a function of the zinc-coordination center of Shh for the association of Shh to the ECM and non-cell autonomous signaling. The zinc coordination domain of Shh appears to be involved as μM amounts of zinc are required for ECM association, while mutants in the zinc coordination domain are insensitive to zinc in the medium and have reduced activity in non-cell autonomous signaling. The observation that Shh-E177A is unable to mediate signaling from the notochord to the overlying neural tube (a textbook example of non-cell autonomous Shh signaling), but is fully capable to induce the Hh response when expressed in the developing neural tube (likely cell-autonomously) [7], provides in vivo evidence that the zinc-coordination domain of Shh is required for non-cell autonomous signaling, although not for the productive binding of Shh to its cognate receptors. The initial experiments that demonstrated that E177 is dispensable for the activation of the Hh response is easily explained as this mutant ligand was added to the responding cells as a purified and soluble fraction [4], thus bypassing the requirement for the function of the zinc-coordination domain. This is further supported by structural analysis of the Ptch1/Shh complex demonstrating that the extreme N-terminus of Shh interacts with Ptch1 [10–12] and suffices to alter Ptch1 activity [13]. These observations further demonstrate the dispensability of the zinc-coordination domain to activate the Shh response in the responding cell.

### Do bacterial Hhs and Shh share a peptidase activity?

Our observations indicate that Shh distribution away from the sites of synthesis and non-cell autonomous Shh signaling can be enhanced under low-calcium and high zinc conditions. The surprising sequence similarity between bacterial and mammalian Hedgehog proteins strongly suggest they have similar functions. It is striking that the conservation between BacHhs and animal Hhs is much greater than between Hedglings and Hhs (Fig. S1 A,B,D), with mollusk, echinoderm and cephalochordate Hedglets even less conserved (Fig. S1 A). The conservation between BacHhs and animal Hhs, but not Hedglings and Hedglets, includes all residues critical for catalysis and calcium binding further supporting the notion that both are calcium regulated peptidases. The organization of bacterial genomes into operons helps in the assignment of possible functions of unknown proteins. The putative role of BacHh (as a M15A peptidase [37]) in the modification of the bacterial peptidoglycans is further supported by the observation that in *Mesorhizobium* and *Bradyrhizobium* the *BacHh* gene is surrounded by genes (likely constituting an operon) that code for proteases, including lysozyme, N-acetylmuramoyl-L-alanine amidase, a peptidoglycan endopeptidase (peptidase M23A), several Trypsin homologs (peptidase S1), a zinc Matrix Metalloprotease (MMP) homolog (peptidase M54), an endonuclease, peptidase S53, and possibly a Phytase (DUF3616). This complex of enzymes might be involved in bacterial feeding or scavenging. BacHhs in *Rhizobiacea* are not part of the core genome [38], as the majority of these bacteria do not carry *BacHh*, a further indication that BacHhs provide a niche-specific specialized function. It remains plausible that this niche function in bacteria was useful in animal development after a gene transfer event into a *cnidarian* ancestor. Hedgling distribution in *porifera* and *choanoflagellates* [39] indicates that they are phylogenetically older, while the curious distribution of Hedglets in mollusks and basal deuterostomes remains enigmatic. Still the loss of catalytically important residues in Hedglings and Hedglets further emphasize that Hhs share a catalytic function with (the likely ancestral) BacHhs.

Possible mechanisms of catalysis of zinc peptidases have been elucidated with the help of structural models of enzyme-inhibitor complexes. Thermolysin is a well-studied zinc metallopeptidase structurally related to Shh [3, 14]. Shh and Thermolysin coordinate zinc via two histidine and an aspartic acid residue. A catalytic glutamic acid residue initiates catalysis (E177 in mouse Shh) by accepting a proton from water to form the nucleophilic hydroxide that attacks the carbonyl carbon, further stabilized by the coordinated zinc. With the two stacking histidine (or occasionally tyrosine) residues a pentacoordinate transition state is formed and resolved into the hydrolyzation products [40, 41].

M15 peptidases cleave peptidoglycans, the major component of the bacterial periplasmic space, and a major component of detritus. Peptidoglycans are analogs of proteoglycans that are common in extracellular matrix (ECM) of animals. Therefore, it is possible that functional conservation between BacHhs and Shh is reflected in the ability of Shh to cleave or modify proteoglycans, thus affecting the Shh response and/or distribution, independent of its binding to the canonical Hh receptors. Although any Shh antagonist could be a possible target for the putative Shh peptidase activity, the Hh-interacting protein (Hhip) is an unlikely substrate candidate as it binds to Shh via the zinc ion, thereby replacing the catalytic water. This mode of binding is akin to that of a metalloprotease/inhibitor interaction [5], and thus likely to inhibit the putative catalytic function of Shh instead of being a substrate. Still, it leaves open the possibility that the main mechanism by which Hhip inhibits Hh signaling is not only via ligand sequestration [42, 43], but actually by repressing the peptidase activity of Shh.

### Are Hhs proteoglycan peptidases?

Hedglings and Hedglets are related to Hhs, and the conserved domains possibly homologous. All animals that have Hedglet also have a *Hh* gene and it is thus plausible that Hedglets are derived from Hh. Hhs are not found in any eukaryote except *cnidarians* and bilaterians. The distribution of Hedgling and Hhs only overlaps in *cnidarians*, but Hedgling can also be found in sponges and choanoflagellates (Fig. S1). This suggests two evolutionary events giving rise to these proteins; one occurring in a *Choanoflagellate* ancestor that originated the gene coding for Hedgling, and an independent event in a *Cnidarian* ancestor that gave rise to modern Hh. The absence of both Hedgling and Hh from algae, plants, fungi, in addition to almost all unicellular eukaryotes makes it unlikely that both Hh and Hedgling linearly evolved from a BacHh protein that could have been present in the Ur-eukaryote, but more likely are products of more recent gene transfers from bacteria. The distribution among eukaryotes of Glypicans and Hs is overlapping, and both are first observed in *Cnidarians* and present in all bilaterians. A more recent evolutionary relationship between BacHh and Hhs is further supported by the observation that the C-terminal residue of many BacHhs perfectly aligns with the exon 2 splice donor site in *Hh* genes, thereby providing a parsimonious explanation how a *BacHh* gene was incorporated in a eukaryotic genome giving rise to *Hh*. This is in contrast to the much less conserved Hh domain in Hedgling that is encoded within a single large exon. Given the central role of Gpcs in the distribution of and response to Hhs (including Shh), Glypicans and Hhs might have co-evolved possibly as a peptidase/substrate combination, co-opting the peptidoglycan activity of BacHhs to cleave the proteoglycan Glypican. Shh binds heparan sulfate (HS), the O-linked glycosaminoglycan sidechain of Gpcs and other proteoglycans [44], via the N-terminal Cardin-Weintraub motif, which plays a crucial role in the release of Shh from the producing cell [24]. Although HS is thought to merely aid in scaffolding of a release complex [45, 46], our results could hint to an additional role for HS in guiding Shh to its peptidase target. We cannot exclude that at least part of the signal detected in the Sypro-ruby staining of the ECM extract of *ShhN*-transfected cells (Fig. 1a) is in fact cleaved substrate.

Hh-like bacterial peptidases (M15A) are predicted to be carboxy (trans) peptidases, cleaving adjacent to the D-ala that is linked to the murein glycans [47, 48]. By analogy, Shh might cleave an unusually modified C-terminal residue. It is intriguing that the C-termini of Glypicans are linked to the GPI anchor that restricts them to the cell surface [49]. Solubilization of Shh-sequestering glypicans by GPI removal would elegantly reconcile the observed peptidase-dependent entry of Shh into the ECM with the important effects of glypicans on Shh signaling and distribution. If Shh remains attached to its potential substrate after cleavage and enters the ECM in a complex or alone remains unresolved.

### *Drosophila* is the exception

It is perhaps unfortunate that Hh was discovered in *Drosophila,* as of all animals sequenced, only Hh in *Drosophilids* is divergent for two of the three residues that coordinate zinc and has a valine residue at the equivalent position of the critical E177. The predicted lack of peptidase activity in *Drosophilid* Hhs is remarkable and further supports the observation that the putative peptidase activity is not required for the Hh/receptor interaction. Perhaps stricter reliance on cytonemes in *Drosophila* that detect Hh at its source [50] renders the ancestral peptidase activity obsolete. Nevertheless, this loss of the putative peptidase activity is unique to *Drosophilids*, as all other (sequenced) animals retain the typical zinc coordination motif and the associated E177 equivalent that are required for catalytic activity. Based on the loss-of-function of several mutants, this intrinsic property is likely a zinc metallopeptidase activity, just like the bacterial counterparts of Shh. Still, the observation that substitution of the Shh calcium/zinc sequences with those of BacHh results in a protein that does not enter the ECM, indicates that their substrates are not interchangeable.

### Is Shh oligomerization preventing ECM association?

The zinc coordination center of Shh and in particular E177 are disadvantageous for Shh multimerization [7]. Zinc prevents oligomerization and we find that zinc is a potent agonist of ECM association and putative peptidase activity. Furthermore, while the E177A mutant prevents ECM association it enhances oligomerization [7]. These observations are consistent with the idea that the putative peptidase activity of Shh prevents or reverts multimerization. This notion is further supported by the structural observation that the cholesterol-modified C-terminus is in close proximity to the zinc coordination center, raising the hypothesis that Shh could have intermolecular autoproteolytic activity that prevents oligomerization [3]. Indeed, multimer size has been reported to be negatively correlated with long-range signaling activity of Shh [51]. This observation could explain why cholesterol-unmodified Shh-C199*, which would be expected to form multimers less efficiently due to the absence of cholesterol, is more readily detectable in the ECM of transfected Hek293t cells and is an efficient non-cell autonomous ligand. Although an inter- or intramolecular autoproteolytic activity has not been demonstrated for ShhNp, many peptidases are produced as pro-proteins that undergo either an intra- or intermolecular activation event. The intramolecular activation events are often auto-catalyzed by the intrinsic peptidase domain [52]. If Shh has retained these bacterial characteristics it is possible that the autocatalytic de-oligomerization event results in a form of ShhN with a peptidase activity that can modify ECM components. In addition, Shh is subject to further N-terminal processing, or “shedding”, prior or concomitant to its release from the cell [53], but this event can be mediated by a family of zinc-metalloproteases called a disintegrin and metalloprotease (ADAM), in concert with the scaffolding protein Scube2 [54]. These metalloproteases share overlapping functions and are therefore sufficient, but not essential, for Shh shedding [55], hinting at the variety of paths Shh can follow to enter the extracellular space.

Although mutations of the central zinc coordinating triad are unstable and thus cannot be easily assessed for loss of peptidase function, mutations of several other catalytically important residues (E177, H135 and H181) are not destabilized and show a loss in the ability of Shh to enter the ECM. Together with the observation by Himmelstein and colleagues [7] that ShhE177A cannot signal from the notochord to the overlying neural plate strongly supports the idea that a Shh-associated peptidase activity is required for non-cell autonomous signaling by promoting its distribution away from the source cells. The implications of a peptidase function that is intrinsic to Shh for normal, non-cell autonomous signaling are significant. The Zn-coordination domain is found mutated in some individuals with the Shh signaling-related birth defect holoprosencephaly [8, 9], further indicating that the intrinsic peptidase activity of Shh is important for normal function. Furthermore, a catalytic interaction between Shh and HSPGs that affects non-cell autonomous signaling would provide significant new insights in developmental and tumor-inducing mechanisms, including the roles of Ext1 and Ext2 as tumor suppressors [56], roles of Gpcs as tumor suppressors [57, 58] a role for extracellular calcium as an inhibitor of Shh that contributes to Shh-mediated morphogenesis, or that affects tumor formation or progression, and a requirement for μM amounts of zinc for Shh activity in general.

Our findings challenge the dogma of the so called “pseudo catalytic domain” of Shh, and we provide a function for the apparent peptidase activity intrinsic to ShhN. None of our observations are in conflict with previous reports, as we confine the activity of the Shh-associated peptidase upstream of the interaction between Shh and its receptors.

## Conclusions

Our observations support the notion that the remarkable sequence similarity between the BacHhs and Shh reflects to a conserved function as a glycopeptidase. All ShhN mutants that are predicted abolish its intrinsic peptidase activity fail to bind to the ECM and have an impaired ability to signal non-cell autonomously. However, BacHh cannot mediate the activities of ShhN, indicating that the substrates for BacHh are not conserved in metazoans.

## Methods

### Sequence analysis

Bacterial Hedgehogs, Hedglings and Hedglets were identified via protein-protein BLAST (NCBI) and HMMER (ensemble) searches [59] using the peptide sequence of the Shh N-terminal domain as the initial query sequence. Conserved sequences were manually curated to contain only the calcium and zinc coordination motifs (around 105 residues). Sequences (supplementary file) were aligned in Clustal Omega (EMBL-EBI). An average distance tree and a PCA plot were generated in Jalview [60], using the BLOSUM62 algorithm. Visualizations of the ShhN structure were generated in UCSF Chimera using Protein Database (PDB) ID 3D1M [61].

### Materials

MG-132 and Concanamycin A were from Calbiochem, Chloroquine and ZnCl_2_ from Sigma, CaCl_2_ from Fisher Scientific, and Dynasore from Abcam.

### Cell culture

*Ptch1*^*−/−*^*;Ptch2*^*−/−*^ fibroblasts were derived from mouse embryonic stem cells received from Allen Bradley (AB1) and are described elsewhere [28]. *Ptch1*^LacZ/LacZ^;*Ptch2*^−/−^;*Boc*^−/−^;*Cdo*^−/−^;*Gas1*^−/−^*;Shh*^−/−^ were derived from *Ptch1*^LacZ/LacZ^;*Ptch2*^−/−^*;Shh*^−/−^ cells and are described elsewhere [28]. No mice were directly involved in this study. Hek293t cells were purchased from ATCC. All cells were cultured in DMEM (Invitrogen) supplemented with 10% FBS (Atlas Biologicals). Mouse embryonic stem cells were cultured in DMEM (Invitrogen) supplemented with 20% FBS (Atlas Biologicals), 2 mM L-Glutamine (Gibco), 1X MEM non-essential amino acids (Gibco), 0.001% 2-Mercaptoethanol (Gibco), and Leukemia Inhibitory Factor (LIF) titrated to optimal growth support (0.001%). Cells were transfected using Lipofectamine2000 reagent (Invitrogen) according to the manufacturer’s protocol.

### DNA constructs

The following mutations were created via site-directed mutagenesis: Shh-C199*/E177V, Shh-C199*/H135A, Shh-C199*/H135Y, Shh-C199*/H181A, Shh-C199*/H181Y, Shh-C199*/H135A/H181A, Shh-C199*/H135Y/H181Y, Shh-C199*/E90A/E91D/D96A/D130N/D132L, Shh-C199*/E90A/E91D/D96A/E127A/D130N/D132L. *Bradyrhizobium paxllaer*i *BacHh* (EnsemblBacteria: LMTR21_38280, NCBI: WP_065756078.1) was codon optimized for eukaryotic expression using the IDT DNA Codon Optimization Tool, ordered as a gBlocks gene fragment from IDT DNA, and cloned into *pcDNA3.1*(+). Both the Shh-C199* vector backbone including the Shh N- and C-terminus as well as the calcium and zinc coordination motifs of *Bradyrhizobium paxllaeri BacHh* were PCR amplified, separated on a 1% agarose gel, and extracted with MinElute columns (QIAGEN). The fragments were cut with *BsaI* and ligated with T4 DNA ligase according to the Golden Gate cloning protocol (New England Biolabs).

### Immunostaining

*Ptch1*^*−/−*^*;Ptch2*^*−/−*^ fibroblasts were plated on 12 mm glass cover slips and transfected with Shh-C199* the following day and subsequently allowed to recover for 24 h. The transfected cells were then incubated for 24 h in serum-free DMEM containing varying concentrations of CaCl_2_ or ZnCl_2_, the cells were detached from the cover slip with PBS. The cover slips were washed with PBS at least 5 times and blocked with 10% heat-inactivated goat serum in PBS with 0.1% TritonX (PBS-T). Mouse α-Shh (5E1, Developmental Studies Hybridoma Bank) was used at 1:30 in blocking solution and goat α-mouse Alexa568 secondary antibody (Invitrogen) at 1:1000 in blocking solution. Shh distribution was visualized with a Zeiss Observer at 10x and 63x magnification.

For live staining, transfected cells were incubated in serum-free medium for 20 h. An α-HA antibody 3F10 (Sigma) was added for another 4 h (1:1000) before cells were fixed with 4% PFA in PBS and blocked in 10% heat-inactivated goat serum in PBS. A goat α-rat Alexa488 secondary antibody (Invitrogen) was used at 1:1000 in blocking solution and nuclei stained with DAPI.

### Western blot/SYPRO ruby staining

Hek293t cells were plated in 12 well plates and transfected with Shh mutants as indicated the next day. Twenty-four hours after transfection, the medium was switched to serum free DMEM with the indicated calcium and zinc concentrations overnight. Cells were then detached from the plate with PBS and lysed in a microcentrifuge tube with RIPA buffer (150 mM NaCl, 50 mM Tris-HCl, 1% Igepal, 0.5% Sodium Deoxycholate, and protease inhibitors). The lysate was incubated for 30 min on ice and cleared by centrifugation. For isolation of ECM-bound Shh, the decellularized tissue culture dish was washed with PBS and deionized water at least 5 times and scraped with a cell scraper and 5X SDS sample buffer heated to 95 °C, as described [23]. A fifth of the sample was run on a 12% SDS-PAGE gel and transferred to a 0.45 μ nitrocellulose membrane. Membranes were blocked in 5% milk in Tris-buffered saline with 0.1% Tween-20 (TBS-T) and incubated with a polyclonal rabbit α-Shh antibody (H2, 1:10,000) [62] in blocking solution, followed by incubation with a goat α-rabbit Alexa647 secondary antibody (Invitrogen, 1:10,000) in blocking solution. GAPDH was used as a loading control (Rabbit α-GAPDH, 14C10, Cell Signaling Technologies). Western Blots were visualized with a ChemiDoc visualization system (Bio-Rad).

Alternatively, the SDS-PAGE gel was stained with SYPRO-Ruby gel stain (Thermo-Fisher) according to the manufacturer’s instructions and visualized with a ChemiDoc visualization system (BioRad).

### Elisa

Hek293t cells were plated in 96 well plates and transfected with Shh-C199* and Shh-C199*/E90A/E91D/D96A/E127A/D130N/D132L in triplicates the next day. Twenty-four hours after transfection, the medium was replaced with DMEM containing 0.18 mM or 1.8 mM Calcium and Zinc concentrations ranging from 0.001 to 1 μM for 48 h. The cells were removed from the plate with PBS and deionized water. The plates were blocked with PBS + 5% heat-inactivated goat serum, incubated with mAB5E1, followed by an HRP conjugated α-mouse secondary antibody (Invitrogen). Western-Lightning Plus-ECL (Perkin Elmer) was added to the wells and luminescence was measured in a Wallac Victor3 plate reader (Perkin Elmer).

### ECM signaling assay

Hek293t cells were plated in 24 well plates and transfected with the indicated Shh-C199* variants the next day. Twenty-four hours after transfection, cells were washed off with PBS and each well washed with PBS extensively to remove residual cells. LightII reporter cells [63] were added and the medium was switched to DMEM as soon as the cells were adherent. Forty-eight hours later, the cells were lysed and luciferase activity was measured using the Dual Luciferase Reporter Assay System (Promega). Firefly luciferase measurements were normalized against Renilla luciferase measurements for each technical replicate to control for differences in cell growth. Firefly/Renilla luciferase values were then normalized to the mock control average for each experiment.

### Non-cell autonomous signaling assays

For the non-cell autonomous signaling assay in chimeric three-dimensional aggregates, Hek293t cells were plated in 12 well plates and transfected with Shh-C199* constructs the next day. Twenty-four hours after transfection, cells were washed off the culture dish with PBS and mixed with four times as many mESC *Ptch1*^LacZ/+^*;Shh*^−/−^ reporter cells in non-treated tissue culture plates. Cells were placed in DFNB medium on a rotating platform at 1 Hz for 48 h and 2 μM Retinoic Acid (RA) was added for another 48 h. The cells were then collected, washed once in PBS, and lysed in 100 mM potassium phosphate, pH 7.8, 0.2% Triton X-100. The lysates were cleared by centrifugation and 20 μl analyzed in triplicates for Ptch1:LacZ expression using the Galacto-Light chemiluminescent kit (Applied Biosciences). Lysates were normalized for total protein using the Bradford reagent (BioRad).

For the long-range signaling assay, Hek293t cells were plated in 12 well plates and transfected with the indicated Shh-C199* constructs the next day. Twenty-four hours after transfection, cells were washed off with PBS and collected in a conical tube. Aggregates were allowed to form for 48 h in DFNB medium in non-treated tissue culture plates rotated at 1 Hz. Similarly, four times as many mESC *Ptch1*^LacZ/+^*;Shh*^−/−^ reporter cells as Hek293t cells were aggregated in DFNB medium for 48 h rotated at 1 Hz. Hek293t aggregates and 2 μM Retinoic Acid (RA) were added to the mESC organoids for another 48 h. Ptch1:LacZ expression was measured as described above.

### Genome editing

*Ptch1*^LacZ/LacZ^;*Ptch2*^−/−^;*Boc*^−/−^;*Cdo*^−/−^;*Gas1*^−/−^*;Shh*^*−/−*^ were derived from *Ptch1*^LacZ/LacZ^;*Ptch2*^−/−−^*;Shh*^*−/−*^ cells [64]. TALEN constructs targeting the first exon of mouse *Cdo* and *Gas1* were designed and cloned into the pCTIG expression vectors containing IRES puromycin and IRES hygromycin selectable markers [65]. The following repeat variable domain sequences were generated: Cdo, 5′ TALEN: NN HD NI NG HD HD NI NN NI HD HD NG HD NN NN; 3′ TALEN: HD NI HD NI NI NN NI NI HD NI NG NI HD NI NN; Gas1, 5′ TALEN: NN NI NN NN NI HD NN HD HD HD NI NG NN HD HD; 3′ TALEN: NN NN NI NI NI NI NN NG NG NG NN NG HD HD NN NI. Two CRISPR constructs targeting a double strand break flanking the first exon of mouse Boc were cloned into pSpCas9 vector with an IRES puromycin selectable marker [66]. The Boc CRISPRs targeted the following forward genomic sequences (PAM sequences underlined): Upstream of first exon 5′ CCTGTCCTCGCTGTTGGTCCCTA 3′; Downstream of first exon 5′ CCCACAGACTCGCTGAAGAGCTC 3′. *Ptch1*^*LacZ/LacZ*^*;Ptch2*^*−/−*^*;Shh*^*−/−*^ mouse embryonic stem cells [64] were transfected with 6 genome editing plasmid. One day after transfection, ES medium with 100 μg/mL hygromycin and 0.5 μg/mL puromycin was added for 4 days. Surviving mESC colonies were isolated, expanded and genotyped by sequence PCR products spanning TALEN and CRISPR-binding sites. PCR screening was performed on cell lysates using primers flanking the TALEN or CRISPR binding sites for the *Boc*, *Cdo*, and *Gas1* loci. *Boc*, (5′) CATCTAACAGCGTTGTCCAACAATG and (3′) CAAGGTGGTATTGTCCGGATC; *Cdo*, (5′) CACTTCAGTGTGATCTCCAG and (3′) CCTTGAACTCACAGAGATTCG; Gas1, (5′) ATGCCAGAGCTGCGAAGTGCTA and (3′) AGCGCCTGCCAGCAGATGAG. PCR products were sequenced to confirm allele sequences. A *Ptch1*^LacZ/LacZ^;*Ptch2*^−/−^;*Boc*^−/−^;*Cdo*^−/−^;*Gas1*^−/−^*;Shh*^*−/−*^ mESC clone was identified harboring a 50 bp deletion in Cdo exon 1, a heteroallelic 480 bp insertion and a 200 bp deletion in Gas1 exon1 resulting in a premature stop codon in the reading frame, and a 450 bp deletion of Boc exon 1. These cells were transfected with *LargeT* and *myc*, and deprived of Lif to generate immortalized fibroblasts.

### Data analysis

Single Factor ANOVA was used to analyze more than two conditions, followed by a Student’s *t*-test with a two-tailed distribution assuming unequal variance comparing two conditions. *p < 0.05, **p < 0.01, ***p < 0.001, ****p < 0.0001.

## Supplementary Information

**Additional file 1.**

## Data Availability

The sequences used for phylogenetic analyses can be found in the supplementary information file. The datasets used and/or analyzed during the current study available from the corresponding author on reasonable request.
