# Supplementary material for: Association of Sonic Hedgehog with the extracellular matrix requires its zinc-coordination center
Source: BMC Mol Cell Biol. 2021 Apr 16;22:22. doi: 10.1186/s12860-021-00359-5 (PMC8052667; doi:10.1186/s12860-021-00359-5)
Supplement: Supplementary file 1 — Additional file 1. [file 12860_2021_359_MOESM1_ESM.docx]

**Association of Sonic Hedgehog with the extracellular matrix requires its zinc-coordination center**

Supplemental Figures

Carina Jägers and Henk Roelink*

University of California, Berkeley, United States

*corresponding author: roelink@berkeley.edu

Short title:

The putative peptidase function of Shh

**Supplementary Figure 1**

**
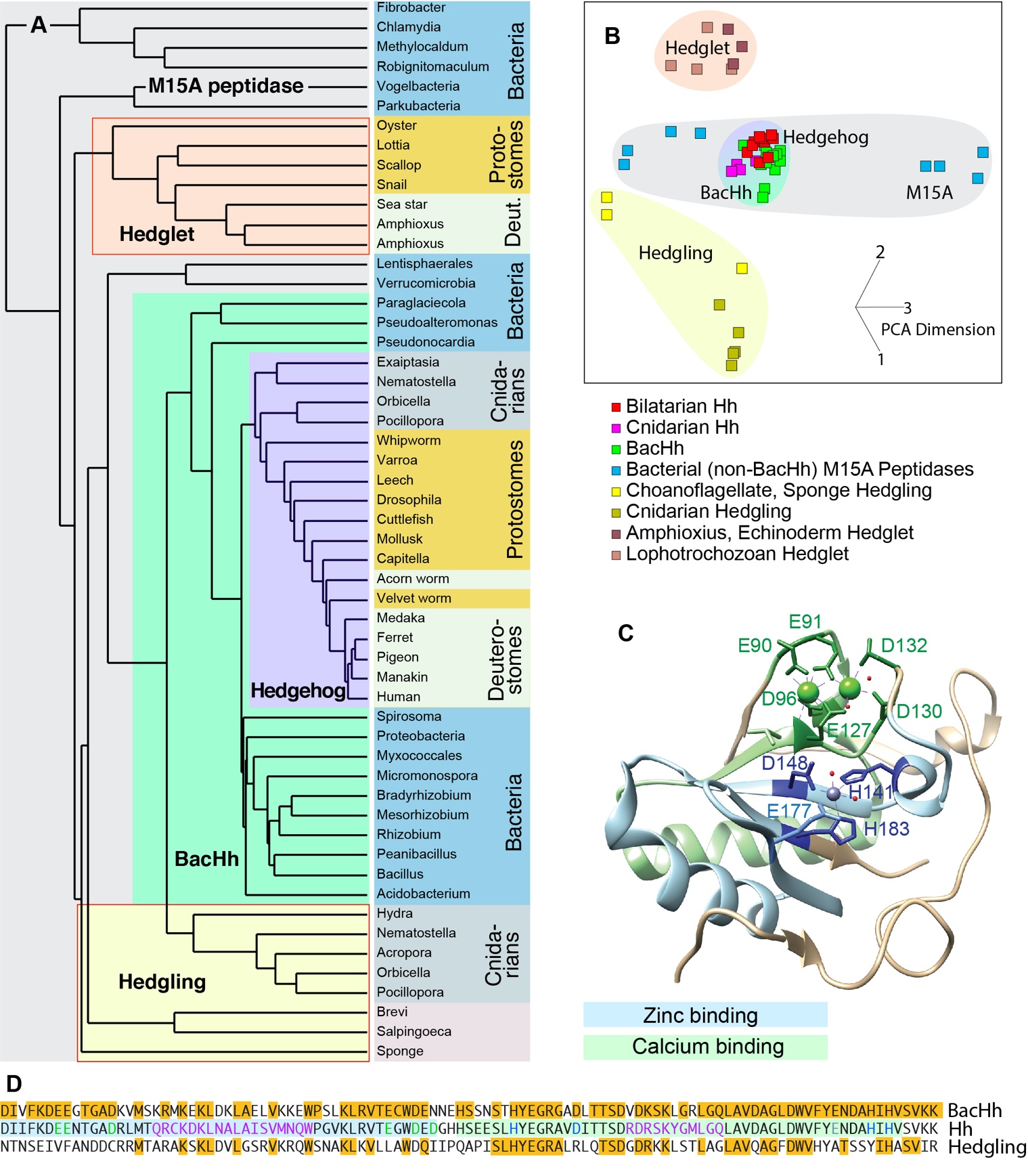
**

**Supplementary Figure 1**. Sequence similarities between BacHhs, Hhs, Hedglings, and Hedglets.

**A:** a similarity tree of Hh sequences encompassing the calcium and zinc coordinating motifs. All Hhs are closely related and root from within the BacHhs. Hedglings (present in Choanoflagellates, Sponges, and Cnidarians) and Hedglets (present in Lophotrochozoans, Amphioxus and Echinoderms) form outgroups, but all are related to bacterial M15 peptidases. Organisms in the same phylum or clade are color coded, and common names of the organisms are used. The sequences used, their accession numbers, and numbering of aligned residues can be found in this supplemental file.  Hedglings and Hedglets (red borders) are predicted to be unable to mediate catalysis. **B:** PCA plot of the tree presented in A. **C:** Structure of Shh with salient residues and domains indicated. **D:** Sequence lineup of the conserved domains of Mesorhizobium BacHh (ESZ55121.1), human Dhh (NP_066382.1), and Choanoflagellate Hedgling (XP_001749037.1). Identical residues are indicated in amber. Salient residues are color coded and color coordinated with C.

**Supplementary Figure 1 Sequences.** Below are the sequences used to generate the tree and PCA plot shown in Supplementary Figure 1. Accession number and stretch of residues used in the lineups are indicated.

>Monosiga brevicollis, [XP_001749037.1](https://www.ncbi.nlm.nih.gov/protein/XP_001749037.1?report=genbank&log$=protalign&blast_rank=1&RID=YATTXZG5014), 354-458

GLSFAPSSGNMYPNVVSARMASRLKVLANLVPRVFGESAAVLVLDAYRAAPLVAAEATLHNTGRAALLTVINVTASLDDELAALASVCADAGFDYVLYNSSAAIY

>Human, [BAA33523.2](http://www.ncbi.nlm.nih.gov/protein/BAA33523.2/), 89-193

IIFKDEENTGADRLMTQRCKDRLNSLAISVMNQWPGVKLRVTEGWDEDGHHSEESLHYEGRAVDITTSDRDRNKYGLLARLAVEAGFDWVYYESKAHVHCSVKSE

>Mesorhizobium sp. L103C131B0, [ESZ55121.1](http://www.ncbi.nlm.nih.gov/protein/ESZ55121.1/), 173-277

IVFKDEENTGADRMMTPRLKSKLDSLANVVASEWPGAKLRVTEAWDEDNEHADASLHYEGRAADLTTNPVDGAKLGRLARLAVDAGCDWVFFEDSSHIHVSVKAG

>Micromonospora sp. HK10, [WP_082159544.1](https://www.ncbi.nlm.nih.gov/protein/WP_082159544.1?report=genbank&log$=protalign&blast_rank=1&RID=YAUEA0ME014), 1063-1166

IVFKDEEKTDADRMMTPRLRDMVNELAALVVKEWPGKKLRVTEGWDENNEHTAESTHYEGRAVDMTVSDLDAAKLGRLARLAVDAGFDWVFYENALHVHASVKK

>Nematostella1 vectensis [XP_001635678.1](https://www.ncbi.nlm.nih.gov/protein/XP_001635678.1?report=genbank&log$=protalign&blast_rank=1&RID=YAUH6RWA014), 84-187

IVFKDEERTGADRLMSKRCREKLRNLATKVKQKWKGVKLRVTEAWDEDGQHSLDSLHYEGRAVDISTSDKDPKKLPDLGSLAVDAGFDWVYYDRRSSIHASVRS

>Nematostella2 vectensis, [ABX84114.1](https://www.ncbi.nlm.nih.gov/protein/ABX84114.1?report=genbank&log$=protalign&blast_rank=1&RID=YAUR1M6801N), 69-171

EVVFENDDCRRTTARAKSKLDVLASRVRQEWAGRKLKVIKAWTDQRTAQDPASLHYEGRALRLQLDNNDRSMLSRLAGLALASGFDWVSYPLNSDYIHASVIRA

>Pseudoalteromonas piratica, [WP_040135141.1](https://www.ncbi.nlm.nih.gov/protein/WP_040135141.1?report=genbank&log$=protalign&blast_rank=1&RID=YAUK8ZN7014), 60-162

PVFKFEEGNFTDVQASEKLCAAIMDLNKLVMKEWPGKTLRVTEAYDQDGEHAKFSLHNEGRAADMTVSDRDLKKLGRLGFLATKAGFSWVYYEHNHIHASVKR

>Spirosoma aerolatum, [WP_080055297.1](https://www.ncbi.nlm.nih.gov/protein/WP_080055297.1?report=genbank&log$=protalign&blast_rank=1&RID=YAV9FHFD014), 211-317

VVFKNEEGDGSDRMMTPVLKTHVDRLADLVRSEWGAGVSLRVTEAWDDTGEHSSSHSLHYEGRAVDLTTSDLDKSKLGRLGRLAVDAGFNWVYYENLLHIHASVTKA

>Varroa destructor, [XP_022667503.1](https://www.ncbi.nlm.nih.gov/protein/XP_022667503.1?report=genbank&log$=protalign&blast_rank=1&RID=YAVCS208014), 83-186

IRFLDDEGTGADRIMTQRCRDKLDTLAVSVMTQWPGVKLRVIESWDEYSHHKSGSLHYEGRAVDFTTDDRHQAKYGMLARLAVEAGFDWVYYETKRHVHASVKP

>Drosophila melanogaster, [NP_001034065.1](https://www.ncbi.nlm.nih.gov/protein/NP_001034065.1?report=genbank&log$=protalign&blast_rank=5&RID=YAVFC86H014), 144-247

ILFRDEEGTGADRLMSKRCKEKLNVLAYSVMNEWPGIRLLVTESWDEDYHHGQESLHYEGRAVTIATSDRDQSKYGMLARLAVEAGFDWVSYVSRRHIYCSVKS

>Crocodylus porosus,[XP_019386078.1](https://www.ncbi.nlm.nih.gov/protein/XP_019386078.1?report=genbank&log$=protalign&blast_rank=18&RID=YAVHYJSK014), 87-190

IIFKDEENTGADRLMTQRCKDKLNALAISVMNQWPGVKLRVTEGWDEDGHHSEESLHYEGRAVDITTSDRDRSKYGMLARLAVEAGFDWVYYESKAHIHCSVKA

>Amphimedon queenslandica, [ABX90059.1](https://www.ncbi.nlm.nih.gov/protein/ABX90059.1?report=genbank&log$=protalign&blast_rank=1&RID=YAVSJC46014), 74-180

ssqatylhfassdcrimssrlytrlsslaeayywryhikilvlkawtpypdysldntslhyegrsvrihvtsrnvtrllkmavsagfdwvmydkkgyarmsvipdac

>Salpingoeca rosetta, [XP_004997926.1](https://www.ncbi.nlm.nih.gov/protein/XP_004997926.1?report=genbank&log$=protalign&blast_rank=1&RID=YAVV8VF2014), 540-646

TVKPDPPTSNGDPSVMSKRLRRHITTLASVVRGVFGDDAYVRVLEAYVEPPADISKASLHNVGRAARITIEGVPDDFASDRLGVLGGLAVEAGFDYVAYTSRDSLYV

>Orbicella1 faveolata, [XP_020616832.1](https://www.ncbi.nlm.nih.gov/protein/XP_020616832.1?report=genbank&log$=protalign&blast_rank=1&RID=YAVZ8MVW014), 68-170

IVFANDDCRRMTARAKSKLDVLGSRVKRQWSNAKLKVLLAWTDQIIPQAPISLHYEGRALRLQTSDGDRKKLSTLAGLAVQAGFDWVHYATSSYIHASVIRDV

>Pocillopora1 damicornis, [XP_027055961.1](https://www.ncbi.nlm.nih.gov/protein/XP_027055961.1?report=genbank&log$=protalign&blast_rank=1&RID=YAW1HW1501N), 66-167

EIDFANDDCRRMTARAKSKLDVLGSTVRRQWSNVKLKVTLAWTDQIMPQAPISLHYEGRAVRLQTSDGDTGKLSTLAGLAVQAGFDWVHYATNSYIHASVIR

>Hydra vulgaris, [XP_004209904.1](https://www.ncbi.nlm.nih.gov/protein/XP_004209904.1?report=genbank&log$=protalign&blast_rank=1&RID=YAW3TT08014), 72-174

IDFQTEDSRLMTSRAKQKIDTLAGLVTTRFGKNMKVNVLKAWTDVVEKEDKLSLHYEGRAFLIRASNNDKKLLSDLMVLAREAGFDWVYYKNEDSIYLSVIPD

>Orbicella2 faveolata, [XP_020632016.1](https://www.ncbi.nlm.nih.gov/protein/XP_020632016.1?report=genbank&log$=protalign&blast_rank=1&RID=YAW63ZG3014), 84-183

IIFRDEEGTGADRLMSKRCKEKLTTLAGLVKGEWPSVKLVVTEAWDEQDQHSPNSLHYEGRAVDLRLSDRDKTKIGLLGRLAVEAGFDWVLYESRSHIHA

>Pocillopora2 damicornis, [XP_027044648.1](https://www.ncbi.nlm.nih.gov/protein/XP_027044648.1?report=genbank&log$=protalign&blast_rank=2&RID=YAWAJX8Y014) 88-190

IIFKDEEGTGADRLMSKRCQDKLNTLADLVRRQWPTVKLVVTEAWDEQGQHSENSLHYEGRAVDLRLSDKDRTKIGYLGRLAVDAGFDWVYYQKRTHIHASVR

>Exaiptasia pallida, [XP_020892909.1](https://www.ncbi.nlm.nih.gov/protein/XP_020892909.1?report=genbank&log$=protalign&blast_rank=1&RID=YAW893ZM014), 87-190

IVFKDEEGTGADRIMSKRLREKLRILAKKVKEKWRGSTRLRVIEAWDEDGTHSAHSLHYEGRAVDITTSDLDKQKYPELGRLAVEAGFDWVFYESQEHIHASVY

>Acidobacteria bacterium, [PYS76727.1](https://www.ncbi.nlm.nih.gov/protein/PYS76727.1?report=genbank&log$=protalign&blast_rank=1&RID=YAWG2D56014), 46-149

IVFKDEEHTGDDRMMTSRLSARVDDLAARVKREFPGLKLRITEAWDDSTIHAPTSRHLEGRAVDITTSDVDHHKLGRLAGLAVEAGFDWVFFENDLHVHASVKK

>Bacillus pseudomycoides, [WP_098188151.1](https://www.ncbi.nlm.nih.gov/protein/WP_098188151.1?report=genbank&log$=protalign&blast_rank=1&RID=YAWJ4UE5014), 989-1092

IQFKDEEGTGADFLMTSRLSDKLNTLAILVNQEWPNIKLRVTEAWDEDNEHSSGSTHYEGRAADITTSDRDGNKLGRLAQLAVDAGFDWVYYENKYHIHVSVKK

>Proteobacteria bacterium, [PZN23661.1](https://www.ncbi.nlm.nih.gov/protein/PZN23661.1?report=genbank&log$=protalign&blast_rank=1&RID=YAWPZ04G01N), 96-197

IVFKDEEATGADLLMTPRLRTRLHELARLVTREWPGVRLRVTEAWDEDSEHGENSIHYEGRAVDVTTSDRDRRKLGRLAGLAIQAGFDWVSHERDHVHASVR

>Vogelbacteria bacterium, [OHA60126.1](https://www.ncbi.nlm.nih.gov/protein/OHA60126.1?report=genbank&log$=protalign&blast_rank=1&RID=YAWUF1K4014), 199-302

ANGYSGINGPGRTDRVHRDVAEATVWVQQNLNSDHNLSTQITAAHTEGVGHSAGSEHYEGRAVDIQPTGGNVTSSNLNIIADYCRQAGFTYVLVENRHVHCDAR

>Methylocaldum marinum, [WP_119628113.1](https://www.ncbi.nlm.nih.gov/protein/WP_119628113.1?report=genbank&log$=protalign&blast_rank=2&RID=YAX161XT01N), 46-148

KSQPPIAVKKGAILAGLDRRMYFALQKARRVWSRYGKLLVVTSGLDGRHKKGSLHYVGLAVDLRSRYFAPSTRRTVTRELRRNLGDEFQVIDEKHHIHVEFDP

>Fibrobacter sp., [WP_143394061.1](https://www.ncbi.nlm.nih.gov/protein/WP_143394061.1?report=genbank&log$=protalign&blast_rank=1&RID=YAX31J8F014), 26-138

NLLIKRVQLKTGVYTGKLDAAMDSAGLVVVAEYHKVMGDSYRPTITSANDYGKHARRSKHYENKALDFRISDVPRNKRSQLVASIRQALGKRFNVFWEEKNTANEHLHIELKE

>Chlamydia trachomatis, [CRH64334.1](https://www.ncbi.nlm.nih.gov/protein/CRH64334.1?report=genbank&log$=protalign&blast_rank=1&RID=YAX73EWC01N), 1-102

MLQFKNNVRLSGVQEEILFIIDRIQRYFEVRLPKRDFVITSLTDGAHMKGSLHPKGLALDMRSRTLDKKEIEYFVTWFRKNFEKSYDLVVEIDHIHIEYDPK

>Parcubacteria group bacterium, [PSO44215.1](https://www.ncbi.nlm.nih.gov/protein/PSO44215.1?report=genbank&log$=protalign&blast_rank=1&RID=YAX8WU5Z01N), 232-331

ASGFRGIAGPGRTGKVKQPWVEKTKQIQEICENRYGGRPFQVTAACTYGVGHSNDSTHYRGEAVDLDPVDATNQQVISCVKEAGGVPYYLDEDSHIHISS

>Robiginitomaculum sp., [PHQ68463.1](https://www.ncbi.nlm.nih.gov/protein/PHQ68463.1?report=genbank&log$=protalign&blast_rank=1&RID=YAXBA8AG014), 158-262

DENDIDIKEGADISDLTDDMTDTFDDISEAWADEAPGVTPVITSGGDGTHSTNSLHYDGNAVDLRTNNLTQAQTTTVASALSTSLGSDYDVVVESDHIHVEYDPG

>Lentisphaerales bacterium, [TFH13511.1](https://www.ncbi.nlm.nih.gov/protein/TFH13511.1?report=genbank&log$=protalign&blast_rank=1&RID=YAXG7KCT014), 91-194

WESDHDGENDEDDHLMHRGVQPLLNQLEKAVASCGAALKVHDASRPSGGGHCATSLHKEGRALDLTADGLTLEDLAKLCWVAGFDWVFNENKRGAEHVHCSSRA

>Paraglaciecola hydrolytica, [WP_068382217.1](https://www.ncbi.nlm.nih.gov/protein/WP_068382217.1?report=genbank&log$=protalign&blast_rank=1&RID=YAXKFD4F01N), 63-166

VVIKFEEGDCSDSKVTKNLKKTIFKLVELIDQEWEGERKLRITEAWDNNAEHTKYSLHNEGRAADITTDDRDTKKLSKLACLAMAAGFSWVKLEKDHVHASVPR

>Rhizobium leguminosarum, [WP_130783679.1](https://www.ncbi.nlm.nih.gov/protein/WP_130783679.1?report=genbank&log$=protalign&blast_rank=1&RID=YAXMXSHR014), 310-413

IVFKDEEGTGADRMMSARLRDGLDRLAAQVGIEWPDVKLRVTEAWDENNEHHGASLHYEGRAADLTTSPRDGDKLGRLGKLAVDAGLDWVFFENSAHIHVSVKR

>Verrucomicrobia bacterium, [HCF95878.1](https://www.ncbi.nlm.nih.gov/protein/HCF95878.1?report=genbank&log$=protalign&blast_rank=1&RID=YAXSMYYP016), 132-232

ESDHDGDWDTENDHLVHRDILPALIRLNALVLQEGATLKIQDAYREEGIHAPASLHREGRALDLTADGMSLARLAQLAVQAGFDWVYYESPKGGGAHIHAS

>Myxococcales bacterium, [RYZ03269.1](https://www.ncbi.nlm.nih.gov/protein/RYZ03269.1?report=genbank&log$=protalign&blast_rank=1&RID=YAXXZ1EH014), 109-212

IVFKDEERNRSDRFMTPRLRRSLVQLSKLVSQTWPKVDLRVTEAWDDRREHGAGSVHYEGRAADITTSDQDPAKLGTLAALAVKAGFDWVFYENATHVHVSVKR

>Branchiostoma1 floridae, [XP_002599309.1](https://www.ncbi.nlm.nih.gov/protein/XP_002599309.1?report=genbank&log$=protalign&blast_rank=1&RID=YAY11YZD01N), 305-414

RMLGSSLDDRCADRVMSKALLDHLRTVQRMVQDEFSGVKLKVLEAWDEPHAGATTGDHPAGSLHYEGRAAKLTLSDGDAAKLPRLAAFCICDGAGYVENKGDHILVAVQK

>Pseudonocardia dioxanivorans, [WP_103381118.1](https://www.ncbi.nlm.nih.gov/protein/WP_103381118.1?report=genbank&log$=protalign&blast_rank=1&RID=YAY4AZE701N), 106-208

VVKDEEGSGADRMMTPRLAELVGVLAAHVAQAFPGRRLRLTEAWDPDGEHSHSSLHYEGRAADLTVDDRDRAKLGRLAALAVQTGFDWVLHENDHVHVSVRAG

>Branchiostoma2 belcheri, [XP_019614930.1](https://www.ncbi.nlm.nih.gov/protein/XP_019614930.1?report=genbank&log$=protalign&blast_rank=1&RID=YAY61MPV01N), 311-413

DRCADRVMTKSMLDLLRKVQKMVKDEFTGVKLKVLEAWDEPHAGATEGDQPAESLHFEGRAAKLTLTDGDTSKLPQLAKNAICAGANFVEHKGDHIFVAVRKQ

>Acanthaster planci, [XP_022111291.1](https://www.ncbi.nlm.nih.gov/protein/XP_022111291.1?report=genbank&log$=protalign&blast_rank=1&RID=YAY8FEHD014), 310-422

HMKGFALNSRCADRTMSARLMATLKTLGKLVSIEWPGVKLLVLEAWDEAHEGSTYTDGDQPAGSLHYEGRAAKLSLSDGDTSKFSRLAGLATCAAADYVEHNGDHIFVAAKKQ

>Sepia bandensis, [ALM01450.1](https://www.ncbi.nlm.nih.gov/protein/ALM01450.1?report=genbank&log$=protalign&blast_rank=1&RID=YAYDYC7J01N), 32-143

IVFRDEESNNEDRMMSKRCKDKLNTLAIAVMNEWPGVKLRVTEAWDTEGHHAPTSLHYEGRAVDITTSDRERSRYGMLARLAVEAGFDWVYYESRSHIHCSVR

>Lottia gigantea, [XP_009064322.1](https://www.ncbi.nlm.nih.gov/protein/XP_009064322.1?report=genbank&log$=protalign&blast_rank=1&RID=YAYFZJSJ014), 299-407

EKPLGNSLNQRCAARLMSQRMYNVLISLQKLVRANGDKLKVEQAFDEKYAGHVADFDATSLYTEGRLVKVTRSVNPSLANYKKLTQWAICSKADFVQNNGDHVLIGVKK

>Crassostrea virginica, [XP_022317995.1](https://www.ncbi.nlm.nih.gov/protein/XP_022317995.1?report=genbank&log$=protalign&blast_rank=1&RID=YAYHEN7N014), 275-379

YPGNYLPNRCAVRVMSPRLFNVLVNLKAYASDANLGGPGGKITVEEAWDGGADPSSLRSEGRMIKVKLSAGNTAANLGKLAQLAICAKADHVSNMGTHLLLSVKK

>Mizuhopecten yessoensis, [XP_021349176.1](https://www.ncbi.nlm.nih.gov/protein/XP_021349176.1?report=genbank&log$=protalign&blast_rank=1&RID=YAYK73W9014), 304-415

GIVGSALSKRCAARTMSYRMYKVINTLQKFVRHNMTLTDKLKVLKAWDEPYADATTGDTSYSRLHTEGRAVVVQLVSSNTASNLEELSHFAICAGADFISHKGDKLEIAVKK

>Bradyrhizobium sp. WSM4349, [WP_018460114.1](https://www.ncbi.nlm.nih.gov/protein/WP_018460114.1?report=genbank&log$=protalign&blast_rank=1&RID=YAYNGH1T014), 210-314

SIVFKDEEGTGADRMMSTRMQAKLDALASLVSAEWPGVKLRVTEAWDENDEHSPTALHYEGRAADITTQPPDGAKLGRLARLAVNAGCDWVFYEDTNHVHVSVKK

>Paenibacillus sp. CAA11, [WP_108465644.1](https://www.ncbi.nlm.nih.gov/protein/WP_108465644.1?report=genbank&log$=protalign&blast_rank=1&RID=YAYRKT04014), 1027-1131

DIVFKDEEGTGADKVMSKRMKEKLDKLAELVKKEWPSLKLRVTECWDENNEHSSNSTHYEGRGADLTTSDVDKSKLGRLGQLAVDAGLDWVFYENDAHIHVSVKK

>Columba livia, [PKK32334.1](https://www.ncbi.nlm.nih.gov/protein/PKK32334.1?report=genbank&log$=protalign&blast_rank=9&RID=YAYU2AFN014), 86-190

DIIFKDEENTGADRLMTQRCKDKLNALAISVMNQWPGVKLRVTEGWDEDGHHSEESLHYEGRAVDITTSDRDRSKYGMLARLAVEAGFDWVYYESKAHIHCSVKA

>Ptychodera flava, [BAR45718.1](https://www.ncbi.nlm.nih.gov/protein/BAR45718.1?report=genbank&log$=protalign&blast_rank=1&RID=YAYVZJ4K014), 88-192

DIIFKDEEGTGADRLMTQRCKDKLNSLAILVMNQWEGIQLRVTEGWDEDGHHAENSLHYEGRAVDITTSDRDKKKYGMLARLAVQAGFDWVYFESKSHVHCSVRS

>Antalis entails, [APD15681.1](https://www.ncbi.nlm.nih.gov/protein/APD15681.1?report=genbank&log$=protalign&blast_rank=1&RID=YAYYEKGY014), 86-190

DVIFKDEEGTGADRMMSKTCRDKLDTLAIFVMNQWTGVKLRVTEAWDEEHHHAKDSLHYEGRAVDVTTSDRDRSKYGMLARLAVNAGFDWVYYESRAHIHCSVNS

>Helobdella robusta, [AAM70491.1](https://www.ncbi.nlm.nih.gov/protein/AAM70491.1?report=genbank&log$=protalign&blast_rank=1&RID=YAZ13NFV014), 270-374

NIIFQNSEGTGADRVMSKRCSDKLNNLASLTMEQWPGVRLRVVEAWDEDETHPEDSLHYEGRAVDVTTSDKDKSKYGMLARLAVEAGFDWVHYEYRSHIHCSVKS

>Oryzias melastigma, [ACL81248.1](https://www.ncbi.nlm.nih.gov/protein/ACL81248.1?report=genbank&log$=protalign&blast_rank=1&RID=YAZ3CKNM01N), 46-150

DIIFKDEENTGADRLMTQRCKDKLNSLAISVMNQWPGVKLRVTEGWDEDGHHFEESLHYEGRAVDITTSDRDKSKYGTLSRLAVEAGFDWVYYESKAHIHCSVKA

>Trichuris suis, [KFD51835.1](https://www.ncbi.nlm.nih.gov/protein/KFD51835.1?report=genbank&log$=protalign&blast_rank=1&RID=YAZ4X53U01N), 95-199

NIVFKDEEGTGADRIMTNRCRYKLNLLALLVSNFWPGVKLRVIDAWEERNRQVVGSLHYEGRAVDITTSDRDNRKIPRLARLAVQAGFDWVYFESRQHVHASVKS

>Capitella teleta, [AAZ04357.1](https://www.ncbi.nlm.nih.gov/protein/AAZ04357.1?report=genbank&log$=protalign&blast_rank=1&RID=YAZ7X5S1014), 82-186

DVVFKDEEGTGADRIMSQRCKDKINTLAISVMNQWPGVKLKVTEAWDEDGFHAKDSLHYEGRAVDITTDDRDRSKYGMLARLAVEAGFDWVYYENRGHIHCSVKS

>Pipra filicauda, [XP_027590561.1](https://www.ncbi.nlm.nih.gov/protein/XP_027590561.1?report=genbank&log$=protalign&blast_rank=1&RID=YAZE8BP6014), 84-188

DIIFKDEENTGADRLMTQRCKDRLNSLAISVMNQWPGVKLRVTEGWDEDGHHSEESLHYEGRAVDITTSDRDRNKYGMLARLAVEAGFDWVYYESKAHIHCSVKS

>Branchiostoma3 floridae, [XP_002607850.1](https://www.ncbi.nlm.nih.gov/protein/XP_002607850.1?report=genbank&log$=protalign&blast_rank=1&RID=YAZMHF8G014), 269-370

HPVGFTPSQRCADRVMSKRLYTALLRVDKHVREQLNARLRITEAWDEPHSGAADGDQAENSLHYEGRAAKLELSGSSDLTSLAKYCICADIDYVEHKGTYLF

>Acropora millepora, [XP_029199742.1](https://www.ncbi.nlm.nih.gov/protein/XP_029199742.1?report=genbank&log$=protalign&blast_rank=2&RID=YAZSBP7A014), 68-170

EIDFANDDCRRMTARAKSKIDVLASRVRGRWSNVRLRVILGWTDQIPVDTQKLLHYEGRALRLQTSDRDSSKLRTLAGLAVEAGFDWVYYASSSYIHASVIRD

>Euperipatoides kanangrensis, [VDH80594.1](https://www.ncbi.nlm.nih.gov/protein/VDH80594.1?report=genbank&log$=protalign&blast_rank=1&RID=YAZVB00R014), 88-190

IIFKDEEGTGADRLMTQRCKEKLNTLAISVMNQWPGIKLRVTEAWDEDNHHSAESLHYEGRAVDITTSDRDRSKYGMLARLAVEAGFDWVFYESRAHIHCSVK

**Supplementary Figure 2**

**
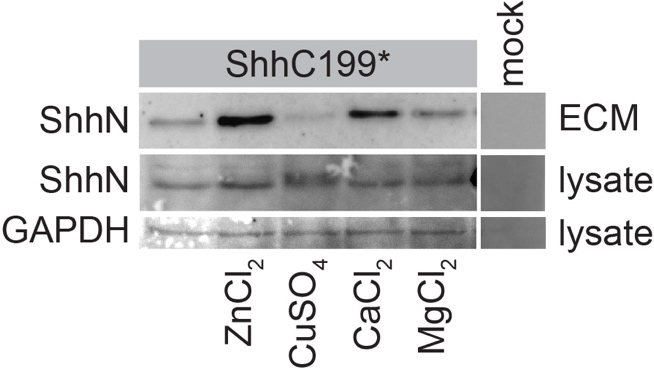
**

**Supplementary Figure 2:** Association of Shh with the ECM is enhanced specifically in response to zinc. Western blot analysis of the lysate and ECM of Hek293t cells transfected with *Shh-C199** and cultured in medium containing 0.18 mM calcium and 5 µM zinc, 5 µM copper, 1.8 mM magnesium or 1.8 mM calcium as indicated.

**Supplementary Figure 3: Uncropped and unedited gel and Western blots for Figure 1:**


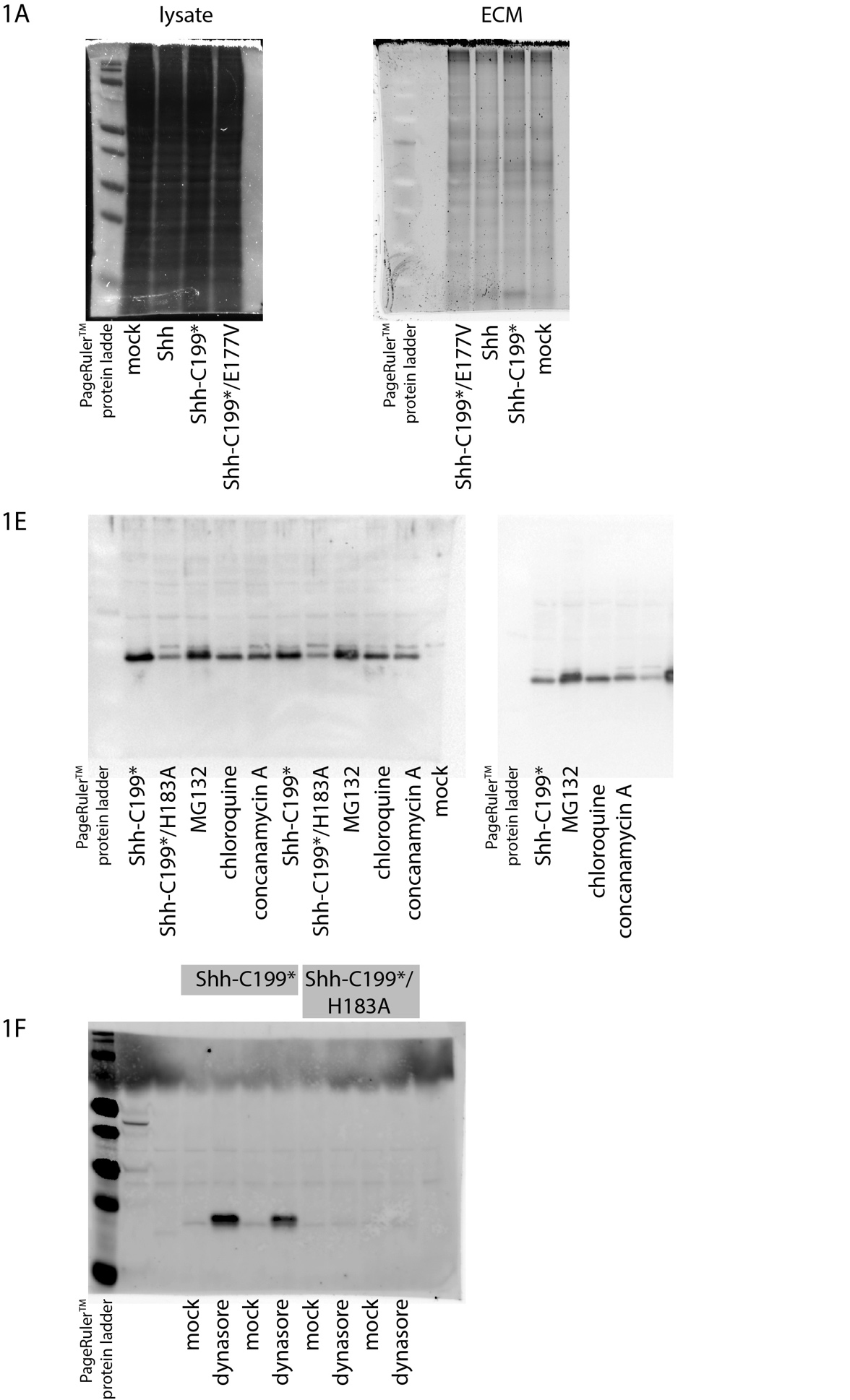


**Supplementary Figure 3: Active ShhN associates with the extracellular matrix.**

**A:** Lysate and ECM deposited by mock and *Shh-C199**-transfected Hek293t cells analyzed by SDS-PAGE and SYPRO-Ruby staining. ShhN is indicated. **E:** Western blot analysis of HEK293t cells transfected with the indicated Shh mutants. 100 nM MG-132 (proteasome inhibitor), 100 nM Chloroquine and 100 nM Concanamycin A (inhibitors of endosome acidification) were assessed for their ability to affect Shh accumulation. **F:** Western blot analysis of HEK293t cells transfected with the indicated Shh mutants, and the effects of the dynamin inhibitor Dynasore (50 µM) was assessed for its effect on Shh accumulation.

M = (PageRuler pre-stained protein ladder)

**Supplementary Figure 4: uncropped and unedited Western blots for Figure 2**


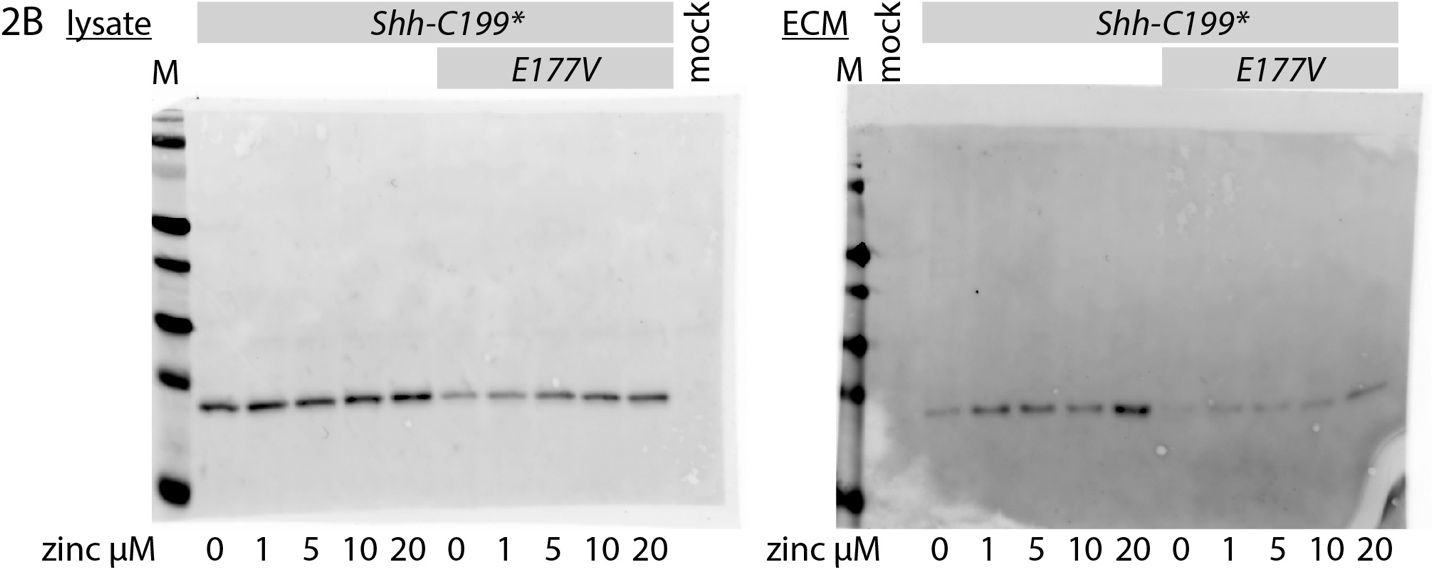


**Supplementary Figure 4: ECM-association of ShhN requires zinc and the catalytic E177V residue.** **B:** Western blot analysis of the lysate and ECM of HEK293t cells transfected with *Shh-C199** and *Shh-C199*/E177V* and cultured in DMEM containing 0.18 mM calcium and the indicated concentrations of zinc.

M = (PageRuler pre-stained protein ladder)

**Supplementary Figure 5: uncropped and unedited Western blots for Figure 3**

**
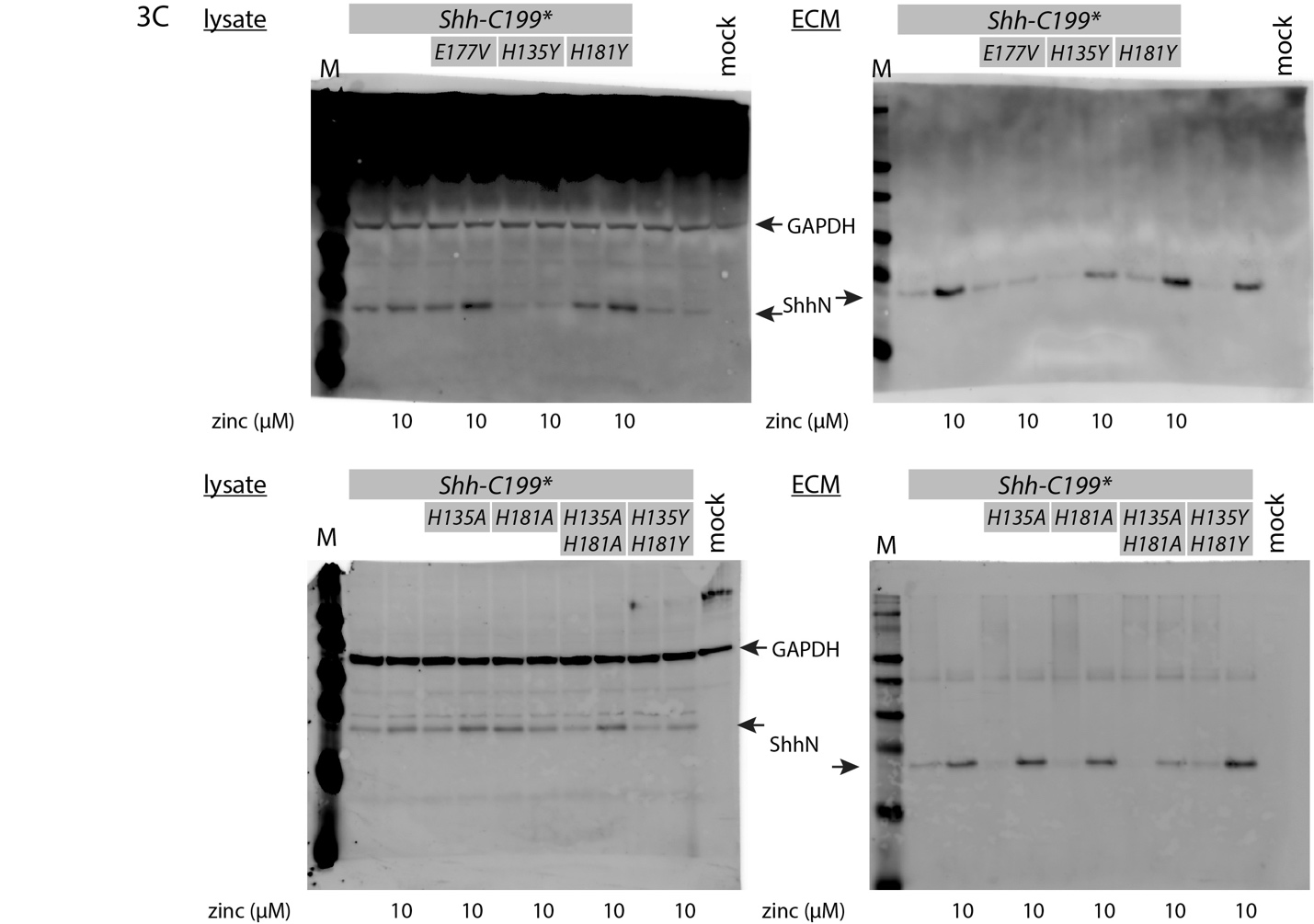
**

**
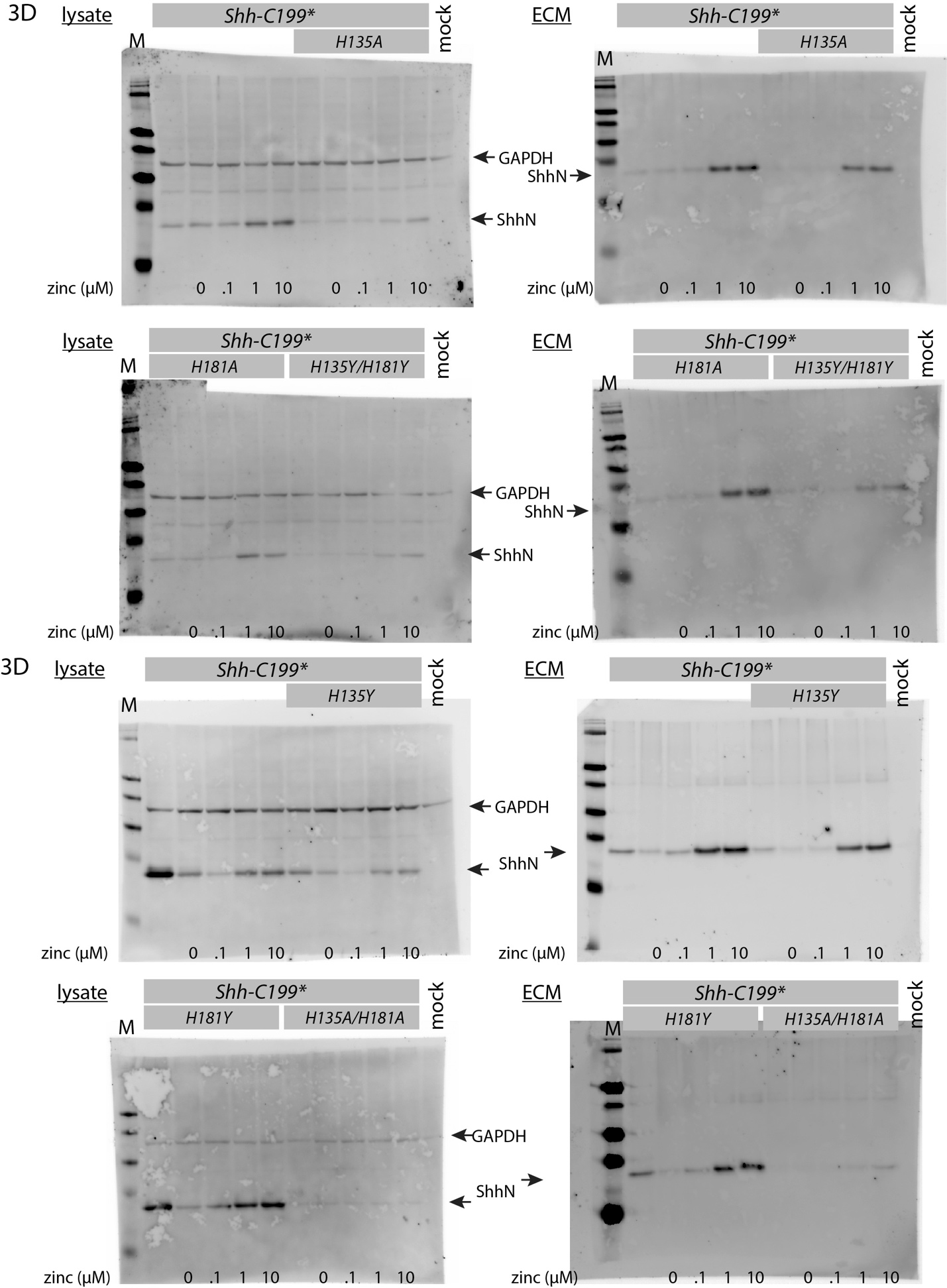
**

**Supplementary Figure 5: Stacking histidines of the zinc coordination center affect ECM association. C:** The effect of mutations of the transition state-stabilizing residues H135 and H181 to alanine (A) or tyrosine (Y) on the zinc-dependent accumulation in the ECM was analyzed on a Western Blot of the extracted ECM from transfected HEK293t cells cultured in 0.18 mM calcium with or without 10 µM zinc. **D:** zinc dose-response analysis of H135 and H181 mutations assessed by Western blot of the lysate and ECM of HEK293t cells transfected with the indicated mutants and cultured in 0.18 mM calcium and increasing concentrations of zinc (0.1, 1, 10 µM). M = (PageRuler pre-stained protein ladder)

**Supplementary Figure 6: uncropped and unedited Western blot for Figure 4**


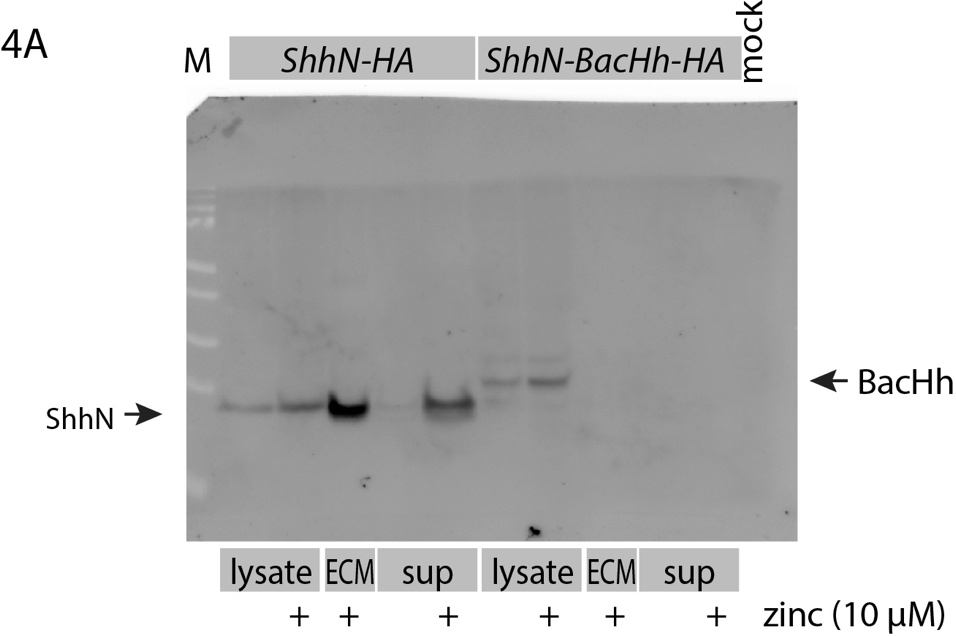


**Figure S4**: **The zinc-coordination domain of BacHh is not sufficient for association with the ECM.** **A:** Western Blot analysis of the lysate, ECM, and supernatant of *ShhN-HA* or *Shh-BacHh-HA* (diagrams) transfected HEK293t cultured in the indicated zinc concentrations.

M = (PageRuler pre-stained protein ladder)

**Supplementary Figure 7, uncropped and unedited Western blots for Figure 5**


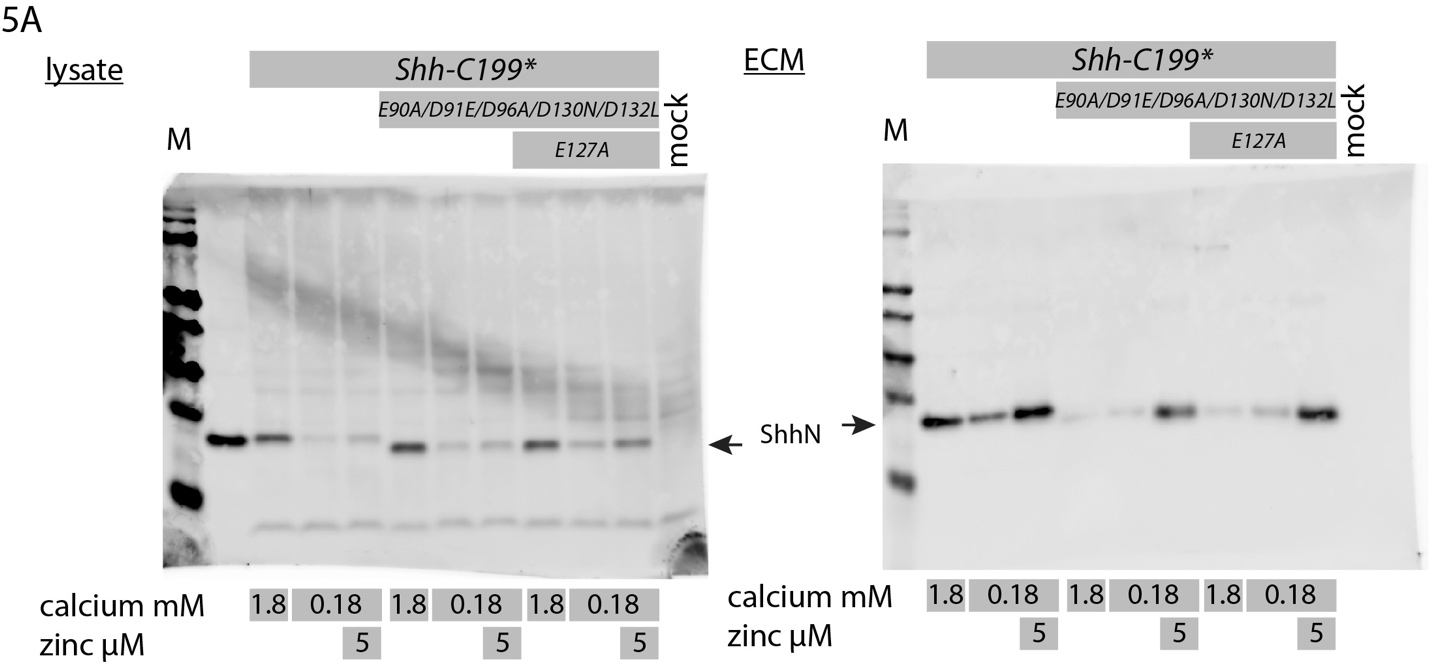


**Figure 5:** Calcium alters the sensitivity of Shh to zinc. **A:** Western blot analysis of lysates and ECM of HEK293t cells transfected with *Shh-C199** and *Shh-C199*/E90A/E91D/D96AD130N/D132L* , or *Shh-C199** and *Shh-C199*/E90A/E91D/D96A/E127A/D130N/D132L* (*Ca^Free^*), cultured in the presence of 0.18 or 1.8 mM calcium, and in the absence or presence of 5 µM added zinc.

M = (PageRuler pre-stained protein ladder)

**Supplementary Figure 8: uncropped and unedited Western blots for Supplementary Figure 2**


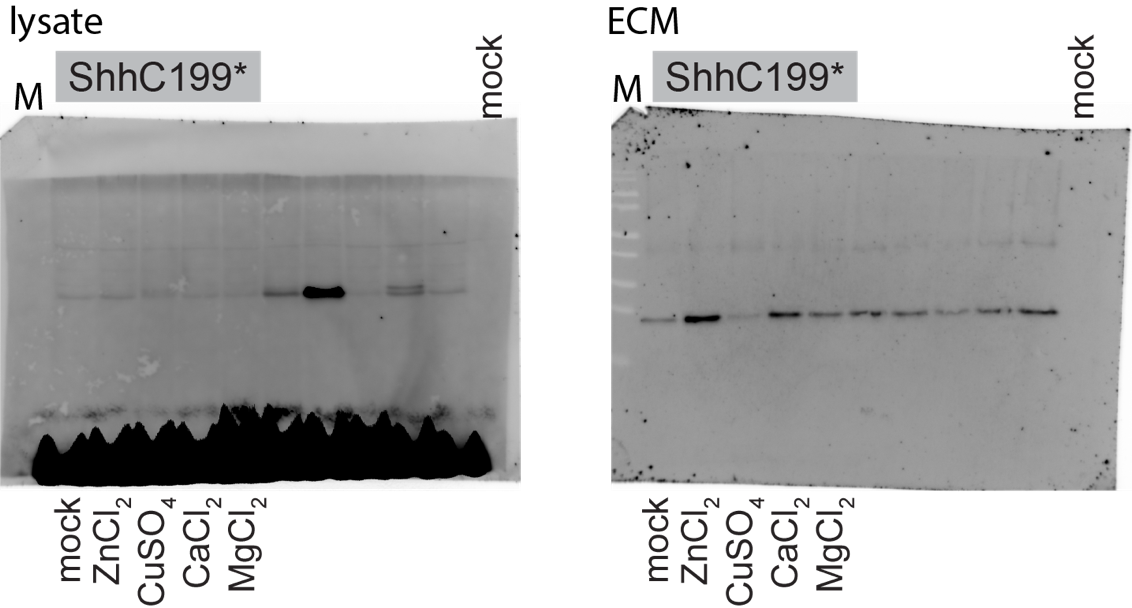


**Figure S8: Association of Shh with the ECM is enhanced specifically in response to zinc.** Western blot analysis of the lysate and ECM of Hek293t cells transfected with *Shh-C199** and cultured in medium containing 0.18 mM calcium and 5 µM zinc, 5 µM copper, 1.8 mM magnesium or 1.8 mM calcium as indicated.
